# Transcriptional analysis of late ripening stages of grapevine berry

**DOI:** 10.1186/1471-2229-11-165

**Published:** 2011-11-18

**Authors:** Sabine Guillaumie, Romain Fouquet, Christian Kappel, Céline Camps, Nancy Terrier, Dominique Moncomble, Jake D Dunlevy, Christopher Davies, Paul K Boss, Serge Delrot

**Affiliations:** 1 Univ. Bordeaux, ISVV, Ecophysiologie et Génomique Fonctionnelle de la Vigne, UMR 1287, F-33140 Villenave d'Ornon, France; 2 INRA, ISVV, Ecophysiologie et Génomique Fonctionnelle de la Vigne, UMR 1287, F-33140 Villenave d'Ornon, France; 3 INRA, UMR1083 Science Pour l'Oenologie, 2 Place Viala, 34060 Montpellier, Cedex 01, France; 4 Comité Interprofessionel du Vin de Champagne, 5 rue Henri Martin, 51204 Epernay, France; 5 Flinders University of South Australia, School of Biological Science, GPO Box 2100, SA 5001, Australia; 6 CSIRO Plant Industry, Waite Campus, Hartley Grove, PO Box 350, Glen Osmond SA 5064, Australia

## Abstract

**Background:**

The composition of grapevine berry at harvest is a major determinant of wine quality. Optimal oenological maturity of berries is characterized by a high sugar/acidity ratio, high anthocyanin content in the skin, and low astringency. However, harvest time is still mostly determined empirically, based on crude biochemical composition and berry tasting. In this context, it is interesting to identify genes that are expressed/repressed specifically at the late stages of ripening and which may be used as indicators of maturity.

**Results:**

Whole bunches and berries sorted by density were collected in vineyard on Chardonnay (white cultivar) grapevines for two consecutive years at three stages of ripening (7-days before harvest (TH-7), harvest (TH), and 10-days after harvest (TH+10)). Microvinification and sensory analysis indicate that the quality of the wines made from the whole bunches collected at TH-7, TH and TH+10 differed, TH providing the highest quality wines.

In parallel, gene expression was studied with Qiagen/Operon microarrays using two types of samples, i.e. whole bunches and berries sorted by density. Only 12 genes were consistently up- or down-regulated in whole bunches and density sorted berries for the two years studied in Chardonnay. 52 genes were differentially expressed between the TH-7 and TH samples. In order to determine whether these genes followed a similar pattern of expression during the late stages of berry ripening in a red cultivar, nine genes were selected for RT-PCR analysis with Cabernet Sauvignon grown under two different temperature regimes affecting the precocity of ripening. The expression profiles and their relationship to ripening were confirmed in Cabernet Sauvignon for seven genes, encoding a carotenoid cleavage dioxygenase, a galactinol synthase, a late embryogenesis abundant protein, a dirigent-like protein, a histidine kinase receptor, a valencene synthase and a putative S-adenosyl-L-methionine:salicylic acid carboxyl methyltransferase.

**Conclusions:**

This set of up- and down-regulated genes characterize the late stages of berry ripening in the two cultivars studied, and are indirectly linked to wine quality. They might be used directly or indirectly to design immunological, biochemical or molecular tools aimed at the determination of optimal ripening in these cultivars.

## Background

Grapevine (*Vitis vinifera *L.) is a nonclimacteric fruit species used as table fruit, dried raisins, and for vinification (wines) and distillation (liquors). In 2007, eight million hectares of grapevines produced 31 billion bottles of wine from vineyards throughout the world. Between 2003 and 2008, global consumption of wine has increased by 6% (International Organization of Vine and Wine (OIV) statistics). The composition of the grape berry at harvest is a major determinant of wine quality. It depends on the interactions between the genotypes of the rootstock and of the variety with the global environment around the plant and the microenvironment around the berries.

Grape development is divided into three phases i.e. two growth phases separated by a lag phase [1]. The first growth period, also called the herbaceous phase, is characterized by embryo development and cell divisions. During this phase, various solutes (malic and tartaric acids, tannins, hydroxycinnamic acids and aroma compounds) accumulate in the different tissues of the berries [2]. All these compounds are important for wine quality. Tartaric and malic acids determine wine acidity, and hydroxycinnamic acids are precursors of phenolic volatiles. Tannins are responsible for the bitter and astringent taste of red wines.

The phase called véraison is a transition phase characterized by a change of berry skin color, from green to white or red, depending on the variety, by the beginning of berry softening, and by a sudden increase in the rate of sugar accumulation. The end of véraison coincides with the onset of maturation, which represents the second period of berry growth, mainly due to water influx and cell enlargement. The maturation phase is characterized by dramatic changes in berry composition [2]. The concentration of some solutes (e.g. malic acid) which are accumulated during the first growth period, decline on a per-berry basis while the concentrations of other molecules (sugars, anthocyanins) strongly increase. Many aroma and flavor compounds essential for wine typicity are produced at a late stage during grapevine ripening.

Several relatively subjective definitions can be used to characterize grape berry ripeness: physiological, technological, aromatic, polyphenolic and oenological. Physiological ripeness corresponds to the time when the berry is ready to be disseminated for plant sexual reproduction and propagation. Technological maturity is the time point beyond which berries do not accumulate more sugars and do not lose any more acidity. Aromatic maturity is characterized by the optimal concentration of aroma and volatile compounds. Phenolic maturity takes into account the quantitative and qualitative evolution of the berry polyphenols in the skin (anthocyanins and tannins) and seeds (tannins).

However, none of them is really satisfactory because few biochemical markers are available, and ripeness depends on their combination and interactions. The wine growers only consider the oenological maturity in order to determine the optimal date of harvest. The oenological maturity tries to take into account and optimize all the forms of maturity previously described while preserving the desired typicity of wines. Therefore, the grapevine berries harvested at oenological maturity show a high sugar/acidity ratio, high anthocyanin content in the skin, and low astringency. However, harvest time is still mostly determined empirically, based on crude biochemical composition (sugar and acid content, and total polyphenol) and on berry tasting. It is therefore important to understand the physiological and molecular basis of grapevine berry ripening that may lead to oenological maturity.

The availability of the grapevine genome [3,4] has boosted large-scale mRNA expression profiling studies of water and salinity stress [5], berry development and ripening [6-8], resistance against pathogenic fungi [9-11] or control of stilbene accumulation [12] using cDNA or oligonucleotide microarrays.

Several multigenic families control the biosynthesis of molecules involved in the grape berry ripening. They are mostly related to cell-wall composition, sugar and water import, organic acid metabolism and storage, and flavonoid synthesis [7,8]. One of the major difficulties currently faced by the wine growers is the lack of accurate descriptors to predict the physiological state of berries. Even though some researchers have analyzed transcription changes during berry development and ripening [6-8], comprehensive transcript profiling has never been used to investigate the last steps of grapevine ripening in relation to wine organoleptic properties. Thus, the signaling networks involved in regulation of the last stages of berry ripening are still unknown.

The present study describes a detailed analysis of gene expression in Chardonnay berries sampled at three different stages during late ripening. Biochemical analysis of grapevine berries and gustatory appraisals of microvinifications were also made. A limited set of genes were consistently differentially expressed in Chardonnay berries whose different ripening stages resulted in different qualities of wine. The expression profiles of some of these genes were also studied and confirmed in the red cultivar Cabernet Sauvignon. The expression of these candidate genes is clearly altered during the last stages of ripening and thus may be considered as potential indicators of late ripening for both cultivars.

## Results and Discussion

### Characterization of Chardonnay samples

**Figure 1:**
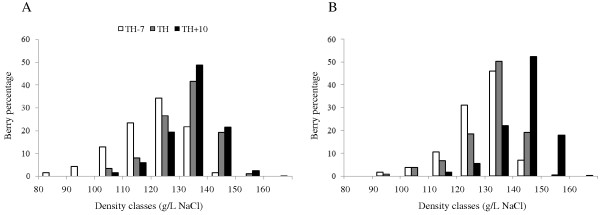
**Representativity of berry classes separated according to their density at three stages of Chardonnay ripening**. In 2005 (A) and 2006 (B), one thousand berries were harvested at each of the three harvest date and were separated into classes according to their density. TH-7, 7-days before theoretical harvest; TH, theoretical harvest; TH+10, 10-days after harvest.

The *Vitis vinifera *cv. Chardonnay berry samples were harvested over the course of berry ripening from the CIVC vineyard in Champagne (France) during fall 2005 and 2006. To take into account the heterogeneity of berry ripening in a vineyard, samples were harvested both as densimetrically sorted berries (DSB) and whole bunch berries (WBB) for better comparison. Samples were collected 7-days before harvest (TH-7), at theoretical harvest (TH) and 10-days after harvest (TH+10). According to DSB, the most representative class was selected for the rest of the study and their density varied from 120 to 150 g/L NaCl (Figure 1). Berry weight, total soluble solids (°BRIX) and potential alcohol content of DSB harvested samples are given in Table 1. The evolution of the mean berry weight of the major DSB class depends on the climate of the year. Mean berry weight remained constant in 2005, whereas it increased in 2006, particularly at the TH+10 stage. According to the CIVC wine-making procedures, the technological maturity corresponded to i) berries free of disease, particularly free of gray mold (*Botrytis cinerea*) and powdery mildew (*Uncinula necator*), ii) a potential alcohol content of 10.0% vol and iii) a total acid content of 8 g H_2_SO_4_/L. In practice, a potential alcohol content higher than 9.0% vol and lower than 12.0% vol (over-ripe) or a total acid content between 6 g H_2_SO_4_/L and 9 g H_2_SO_4_/L can express a high level of the qualitative potential in Champagne wines. The combination and adjustment of the level of these thresholds to the highest quality of wines is based on sensory analysis benchmarks. The potential alcohol and the total acid contents of Chardonnay harvested samples from the CIVC vineyard during fall 2005 and 2006 ranged between 10.19 to 11.60% vol (Table 1) and 5.6 to 8.3 g H_2_SO_4_/L (Table 2) respectively. Therefore, TH-7, TH and TH+10 WBB and DSB samples corresponded to an adequate time span for the study of ripening (Tables 1 and 2).

**Table 1:** Physiological characteristics of densimetrically sorted berries (DSB) of *Vitis vinifera *L. cv. Chardonnay grown in Epernay, France, in the 2005 and 2006 seasons, at three ripening stages.

Harvest date	Density (g/L NaCl)	Berry weight (g)	Total soluble solids (°BRIX)	Potential alcohol (% vol)
09/19/2005 (TH-7)	120 - 130	1.55	18.2	10.19
09/26/2005 (TH)	130 - 140	1.57	19.2	10.86
10/05/2005 (TH+10)	130 - 140	1.55	20.0	11.40
09/14/2006 (TH-7)	130 - 140	1.38	19.3	10.93
09/20/2006 (TH)	130 - 140	1.45	19.2	10.86
10/02/2006 (TH+10)	140 - 150	1.54	20.3	11.60

TH-7, 7-days before theoretical harvest; TH, theoretical harvest; TH+10, 10-days after harvest.

**Table 2:** Physicochemical parameters of microvinifications.

Harvest year	2005						2006					
**Wine stage**	**DMU**			**BW**			**DMU**			**BW**		

**Harvest date**	**TH-7**	**TH**	**TH+10**	**TH-7**	**TH**	**TH+10**	**TH-7**	**TH**	**TH+10**	**TH-7**	**TH**	**TH+10**

Total sugar content (densimetric titration, g/L)	170.0	183.0	193.0	-	-	-	173.0	180.0	192.0	-	-	-
Alcohol content (densimetric titration, % vol)	10.1	10.9	11.4	-	-	-	10.3	10.7	11.4	-	-	-
pH	3.0	3.1	2.9	3.1	3.1	3.2	2.9	3.1	3.1	3.1	3.1	3.2
Total acid content (potentiometric titration, g H_2_SO_4_/L)	7.0	6.2	5.6	5.0	4.6	4.1	8.3	7.3	6.2	5.1	4.7	4.1
Total enzymatic SO_2 _content (Lisa method, mg/L)	13.0	29.0	29.0	33.0	37.0	49.0	34.0	20.0	29.0	37.0	37.0	49.0
Tartaric acid (g/L)	7.0	7.6	6.2	3.7	3.3	2.9	6.8	7.4	8.9	3.5	2.5	2.7
L-malic acid (g/L)	5.3	4.8	3.6	-	-	-	5.5	5.4	5.4	-	-	-
Total sugar: total acid ratio	24.3	29.5	34.5	-	-	-	20.8	24.6	31.0	-	-	-
Total nitrogen (mg N/L)	227.0	261.0	237.0	138.0	203.0	198.0	454.0	223.0	222.0	171.0	197.0	186.0
Ammoniacal nitrogen (Lisa method, mg N/L)	42.0	41.0	59.0	-	-	-	49.0	47.0	34.0	-	-	-
Glucose + fructose (Lisa method, g/L)	-	-	-	0.8	0.9	0.9	-	-	-	0.8	0.8	1.1
Abs atomic potassium (mg/L)	1310.0	1100.0	1480.0	565.0	477.0	484.0	1603.0	1563.0	1823.0	503.0	563.0	502.0
Abs atomic calcium (mg/L)	82.0	57.0	53.0	-	-	-	47.0	99.0	80.0	-	-	-
Serine (%)	7.2	7.1	-	-	-	-	9.8	6.8	-	-	-	-
Threonine (%)	4.5	4.1	-	-	-	-	0.6	0.6	-	-	-	-
Asparagine (%)	0.9	1.0	-	-	-	-	1.1	1.0	-	-	-	-
Glutamine (%)	11.5	8.2	-	-	-	-	19.8	17.1	-	-	-	-
Proline (%)	28.3	36.7	-	-	-	-	26.8	35.5	-	-	-	-
Alanine (%)	19.5	13.9	-	-	-	-	17.8	16.0	-	-	-	-
y-aminobutyric acid (%)	6.4	5.0	-	-	-	-	5.0	5.9	-	-	-	-
Arginine (%)	5.5	4.5	-	-	-	-	9.0	7.6	-	-	-	-

Except proline which is not used by yeast, all amino acids noticed above correspond to 80% of yeast nitrogen needs.

-, not tested; BW, base wine; DMU, decanted must; TH-7, 7-days before theoretical harvest; TH, theoretical harvest; TH+10, 10-days after harvest.

### Microvinification assays and sensory analysis

Microvinification and sensory analyses were done to assess the quality of the wine produced from the berries harvested at the TH-7, TH and TH+10 stages. These analyses were performed to determine whether wines made from the TH-7, TH and TH+10 samples could be discriminated. The overall objective of these combined analyses was to show which harvest time point is the best for producing a quality Champagne wine between the TH-7, TH and TH+10 harvest stages, and thus to associate a transcriptomic profile with the highest wine quality.

The physicochemical parameters determined on the decanted must and base wines for Chardonnay wines are given in Table 2. Similar patterns for total sugar and alcohol contents were found in 2005 and 2006 for decanted must wines derived from TH-7, TH and TH+10 samples. The same was true for the total acid contents in decanted must and base wines. During the ripening process, the sugar and alcohol contents increased in decanted must wines whereas the total acid contents decreased in decanted must and base wines. The sugar to acid ratio is not used in the Champagne area to determine the optimal harvest date, but it is commonly used as a quality index in grapevine [2]. The changes in total sugar/total acid ratio of the Chardonnay decanted musts during grapevine berry late ripening are therefore shown in Table 2. The total sugar/total acid ratio increased during the last stages of ripening process and ranged from 24.5 to 34.5 during fall 2005 and from 20.8 to 31 during fall 2006. At the harvest stage (TH), the total sugar/total acid ratio was different between the decanted must wines derived from samples harvested during fall 2005 and 2006. They varied from 29.5 (TH-2005) to 24.6 (TH-2006). However, and if the 2005 and 2006 vintages are considered as repetitive, an average increase of 16.6% ± 1.6 of the sugar to acid ratio was observed between the TH-7 and TH musts. A similar pattern, i.e. an increase of 17.4% ± 4.2, was also noticed between the TH+10 and TH musts. Thus, a rise of 31.2% ± 2.2 was observed in the overall total sugar/total acid ratio between the TH-7 and TH+10 stages. In contrast, total SO_2_, tartaric and L-malic acids, total nitrogen, ammoniacal nitrogen and calcium contents in decanted must wines and potassium content in base wines showed different trends in 2005 and 2006 vintages. Among the amino acid contents, no difference and consistent evolution was noticed except for proline in decanted must wines of 2005 and 2006 vintages. Proline is not used by yeasts, but is classically high in the Chardonnay cultivar. Among all physicochemical parameters investigated in decanted must and base wines, the relative content in proline (% compared to all amino acids), the sugar, alcohol and total acid contents and consequently the sugar to acid ratio were the only parameters displaying an evolution which can be related to the late ripening progress of Chardonnay berries.

A sensory analysis was performed to distinguish the base wines elaborated with berries harvested at the TH-7, TH and TH+10 stages (Table 3). For each growing season studied, a triangular test was conducted. The data did not reveal any significant difference between the base wines elaborated with the TH-7 and TH berries of the two growing seasons. However, the same comparison between TH and TH+10 or TH-7 and TH+10 base wines indicated significant variations for each year. Sensory analysis demonstrates that wines elaborated from the TH berry samples exhibit typical sensory properties of Champagne wines (Table 3). The TH-7 and TH+10 wines display aromas that are less typical or not typical at all.

**Table 3:** Wine sensory analysis in Chardonnay base wines from 7-days before theoretical harvest (TH-7), theoretical harvest (TH) and 10-days after theoretical harvest (TH+10) samples of the 2005 and 2006 growing seasons.

Triangular tests	Samples	Major sensory descriptors
2005		

TH-7/TH	TH-7	**Slight milk, lively**
	
	TH	**Fruity **(cherry), **round**, **slight bitterness**

TH/TH+10*	TH	**Milk **(yoghurt, toffee, butter), **round**
	
	TH+10	**Reductive character **(sulfur), more **vegetal **than smoked and roasted, **lively**

TH-7/TH+10*	TH-7	**Acid**, **lively**, **aggressive**
	
	TH+10	**Vegetal**, **less acid **versus round and flat mouth, **bitterness**

2006		

TH-7/TH	TH-7	**Acid **(aggressive)
	
	TH	**Reductive hint **(animal), **less acid**

TH/TH+10*	TH	**Reductive character **(cauliflower), **more acid than astringent and bitter**
	
	TH+10	**Reductive character **(hydrocarbon, rubber, burnt wood, vegetable stock versus animal), **acid **(more aggressive), **bitter**, **short**

TH-7/TH+10*	TH-7	**Reductive hint**, **acid **(fresher, harder, aggressive), **aqueous mouth**
	
	TH+10	**Roasted and reductive character **(sulfur, animal, smoke, putrid), **acid **(hard, lively, slight acidity), **round**, **bitter**

Tasting descriptors represent a summary of sensory descriptors employed by a tasting panel of 13 tasters to qualify Chardonnay base wines. *correspond to significant comparison analysis performed (P < 0.05). Bold sensory descriptors are the most representative ones distinguishing two defined base wines.

The global gene expression analysis in the different berry samples (i.e. WBB and DSB) and ripening stages provided us with a fingerprint of the grapevine late ripening transcriptome. In this way, we identified (1) genes that are temporally co-expressed, and (2) individual gene family members that are preferentially expressed in a particular berry sample or ripening stage.

### Differential gene expression in Chardonnay berries during late ripening

Transcriptomic analysis was conducted with the different berry samples (i.e. WBB and DSB). Samples collected at the TH stage were compared to the TH-7 and TH+10 stages respectively in order to emphasize evolutions of gene expression around the TH stage.

**Figure 2:**
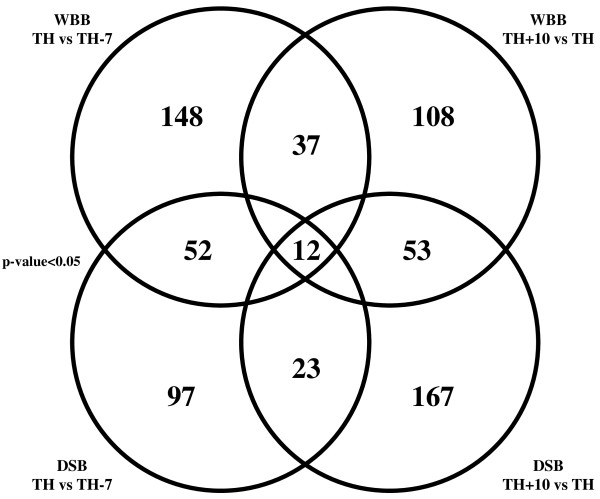
**Venn diagram summary of differentially expressed genes identified in Chardonnay at three stages of ripening**. Chardonnay whole bunches and densimetrically sorted berries were harvested at the 7-days before harvest (TH-7), theoretical harvest (TH) and 10-days after harvest (TH+10) stages during the 2005 and 2006 years. Comparisons of the expression profiles of TH versus TH-7 and TH+10 versus TH were made for whole bunches and densimetrically sorted berries. The total numbers of genes differentially expressed are indicated in respective circles (P < 0.05, ≥1.75-fold). The combined number of genes simultaneously up- or down-regulated is given in intersections between circles. Twelve genes were differentially expressed at all stages of late ripening; some genes were stage or sample type specific while others were overlapping in two stage or sample comparisons (for gene identity, see Tables 4, 5 and 6 and Additional files 1 and 2(Tables S1and S2)). WBB, whole bunch berries; DSB, densimetrically sorted berries.

Among the 14,562 investigated genes, 5 and 7 genes were consistently down-regulated or up-regulated throughout the last steps of grapevine ripening in both WBB and DSB (Figure 2; Table 4) and for the two years studied.

**Table 4:** Differentially expressed genes (P < 0.05, ≥1.75-fold) in Chardonnay grapevine berries all along the investigated ripening periods of the 2005 and 2006 growing seasons.

Putative function	Grape Microarray Accession Number (Vv_#)	Grape Nucleotide Accession Number (mRNA)	Grape Gene Accession Number (GSVIVT#)	Most Homologous *Arabidopsis *Sequence	WBB average ratio (TH vs TH-7)	p-value	WBB average ratio (TH+10 vs TH)	p-value	DSB average ratio (TH vs TH-7)	p-value	DSB average ratio (TH+10 vs TH)	p-value
**Aroma related genes**												

Carotenoid cleavage dioxygenase 4a (VvCCD4a)	Vv_10003015	XM_002268368	GSVIVT01036862001	At4g19170	0.999	2.00E-04	1.219	9.00E-05	1.071	0.00041	1.018	0.00022

**Phenylpropanoid/lignin genes**												

Phenylalanine ammonia lyase (VvPAL1)	Vv_10000977	XM_002281763	GSVIVT01025703001	At2g37040	1.691	6.00E-05	1.076	7.00E-04	1.052	0.00107	1.497	0.00031
Phenylalanine ammonia lyase (VvPAL2)	Vv_10000978	AB015871	GSVIVT01024306001	At3g53260	1.858	0.00013	1.046	0.00069	1.234	0.00025	1.578	0.00024

**Response to dessication**												

Galactinol synthase (VvGolS)	Vv_10000327	XM_002262669	GSVIVT01017634001	At1g56600	-1.307	0.00057	-0.99	0.00142	-1.462	0.00057	-1.107	0.00053
Late embryogenesis abundant protein (VvLEA1)	Vv_10001081	XM_002283966	GSVIVT01033739001	At3g53040	-1.11	3.00E-04	-1.193	0.00118	-1.378	2.00E-04	-1.071	0.00041
Late embryogenesis abundant protein	Vv_10001082	AM474201	GSVIVT01033739001	At3g53040	-1.271	0.00053	-1.3	0.00017	-1.559	0.00016	-1.27	1.00E-04

**Pathogenesis-related genes**												

Pathogenesis-related protein 10	Vv_10010887	XM_002274581	GSVIVT01035059001	-	1.957	0.00178	2.077	0.0022	1.375	0.00037	2.115	0.00055
Dirigent-like protein (VvDIR-like)	Vv_10002588	XM_002285641	GSVIVT01025392001	At3g13650	1.979	0.00087	1.81	0.00053	1.377	0.00456	1.734	4.00E-04

**Hormonal control**												

Histidine kinase receptor (VvHKR)	Vv_10014467	FJ822975	GSVIVT01030060001	At5g35750	-0.943	0.00023	-0.845	0.00031	-0.826	0.00033	-0.94	0.00032

**Unknown function**												

Unknown gene	Vv_10014451	XM_002270095	GSVIVT01010993001	At4g25010	2.263	3.00E-05	0.982	0.00021	1.757	3.00E-05	1.471	6.00E-05
Unknown gene	Vv_10002806	XM_002273032	GSVIVT01038103001	At1g65260	1.375	0.00014	0.884	0.00106	1.01	0.00122	1.095	0.00058
Unknown gene	Vv_10011055	XM_002284158	-	-	-0.849	0.00238	-0.811	0.00091	-0.838	0.00113	-1.032	0.00115

Genes are organized in functional categories. Ratio values are presented as log2. DSB, densimetrically sorted berries; TH-7, 7-days before theoretical harvest; TH, theoretical harvest; TH+10, 10-days after harvest; WBB, whole bunch berries.

These genes belong to five functional categories, including aroma-, dessication- or pathogenesis-related genes and phenylpropanoid metabolism (Table 4). These putative functions were attributed on the basis of homology with grape and *Arabidopsis thaliana *genes. The most homologous *Arabidopsis thaliana *and *Vitis vinifera *genes of each grape oligonucleotide are also indicated in Table 4. Among the 12 genes differentially expressed throughout the last phases of grapevine ripening (TH-7, TH, TH+10), three did not have any known function.

#### Aroma related genes

**Figure 3:**
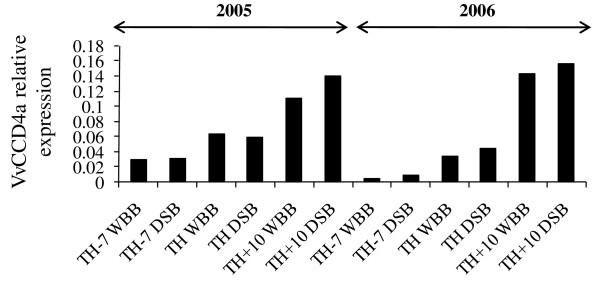
**Bar diagram of *Vitis vinifera carotenoid cleavage dioxygenase 4a *(*VvCCD4a*) transcript abundance: a comparison of qRT-PCR data of Chardonnay whole bunch and densimetrically sorted berries harvested at three ripening stages in 2005 and 2006**. The mRNA level was expressed relative to controls (set at 1), reference gene *EF1-α*. RT-PCR data are reported as means ± SE (error bars) of n = 3 technical replicates. DSB, densimetrically sorted berries; TH-7, 7-days before theoretical harvest; TH, theoretical harvest; TH+10, 10-days after harvest; WBB, whole bunch berries.

Aroma is important for wine quality, and it is therefore interesting that one gene predicted to encode a putative carotenoid cleavage dioxygenase (CCD) was up-regulated during Chardonnay ripening (Table 4 and Figure 3). Indeed, carotenoids are apocarotenoid precursors which play a role in the production of phytohormones (i.e. abscisic acid) and some flavors and aromas. Apocarotenoids are mostly generated by the cleavage of a carotenoid molecule by enzymes of the CCD family [13-15]. Among the carotenoids, the levels of beta-carotene, lutein, flavoxanthin and neoxanthin decrease after véraison in grapevine berries [16]. These carotenoids undergo breakdown reactions that produce C13 norisoprenoid compounds involved in the typical aromas of some grapevine cultivars [17] as was demonstrated with VvCCD1 [18]. The increased transcript abundance of *VvCCD4a *could be related to the presence of apocarotenoids during the end of the ripening process. In grape, four *CCD4 *genes have been identified *in silico *[19], but none has been functionally characterized. According to Huang et al. [20], plants produce at least two different forms of CCD4 enzymes.

Among the CCD4 proteins already characterized for other plants, the *Malus domestica *and *Rosa x damascena *CCD4 proteins (MdCCD4 and RdCCD4) are the closest to VvCCD4a. *In vivo *assays analyzed by SPME-GC-MS showed that MdCCD4 and RdCCD4 cleave *b*-carotene to yield *b*-ionone [20]. However, no cleavage products were found when *MdCCD4 *and *RdCCD4 *genes were co-expressed in *E. coli *strains that accumulated linear carotenoids such as cis-z-carotene or lycopene [20]. We performed isolation and cloning of the *VvCCD4a *gene into pGEX expression vector. In a similar way to various CCD4s such as MdCCD4 or RdCCD4 [20], the co-expression of *VvCCD4a *gene in the strains accumulating cis-*z*-carotene, lycopene, *b*-carotene, and zeaxanthin did not cause a lack of pigmentation in these cultures (data not shown). Although some CCD4 proteins have been shown to cleave carotenoid substrates at the 9,10 and 9',10' positions, they might have different biochemical functions as they may accept different (apo)carotenoids and show various expression profiles.

A subcellular localization study of VvCCD4a protein revealed the chloroplast localization of the VvCCD4a enzyme (data not shown). This is in agreement with the deduced amino acid sequences of all CCD4 proteins, including VvCCD4a, which contain a plastid-targeting transit peptide at the N-terminus [19]. Furthermore, the *Crocus sativus *and *Arabidopsis *CCD4s, have been shown to reside in plastids, where their substrates are localized, suggesting a direct involvement in volatile formation [19]. Altogether, this suggests a potential role for VvCCD4a in berry color, flavor and aroma during late ripening of Chardonnay berries.

#### Phenylpropanoid pathway

Two genes called *VvPAL1 *and *VvPAL2 *encoding phenylalanine ammonia-lyase (PAL; EC 4.3.1.5) were up-regulated throughout the last periods of Chardonnay ripening (Table 4). PAL catalyzes the first step in the phenylpropanoid pathway by removing the NH3 radical from L-Phe to produce trans-cinnamic acid and other phenolic compounds. In grapevine berry, PAL is located in epidermal cells as well as in the seeds [reviewed in [21]]. PAL activity within the grapevine skin is maximal at the first stages of development, and decreases up to véraison. In colored grapevines, PAL activity in the skin shows a second peak after véraison [reviewed in [21]]. There is a close relationship between its activity and the color intensity of colored grapevines [22]. No PAL isoenzyme is detected in the skin of non-colored berries such as Chardonnay during the late ripening [23], nor is there any PAL transcript present [24]. Thus, PAL activity seems to play an essential role in anthocyanin accumulation only in colored grapevine berries. To date, the putative functions of VvPAL1 and VvPAL2 in ripening of white grapevine berries are still unknown. In *Arabidopsis thaliana*, AtPAL1 and AtPAL2 are related to the lignification process [25]. *AtPAL1 *and *AtPAL2 *are responsive to environmental factors like nitrogen depletion or pathogens [26]. Such roles can also be hypothesized for *VvPAL1 *and *VvPAL2*.

#### Response to dessication

Galactinol synthase (GolS; EC 2.4.1.123) is a member of the glycosyl transferase family 8 (GT8) [27] and catalyzes the first committed step in the biosynthesis pathway of raffinose family oligosaccharides (RFOs). GolS synthesizes galactinol, which serves as a donor to form soluble galactosyl-Suc carbohydrates. Accumulation of RFOs is usually associated with abiotic stress such as cold, heat or dehydration [28]. At the protein level, VvGolS (GSVIVP00019670001; Table 4) exhibited 69% identity/78% similarity with *Arabidopsis *GolS1. *AtGolS1 *transcripts were detected during seed maturation and may be implicated in seed osmoprotection [29]. However, RFOs also constitute a significant component of phloem-transported sugars in some plants [30].

Two genes encoding late embryogenesis abundant proteins (LEA) were also down-regulated during the last stages of grape ripening (Table 4). *LEA *expression could be related to the acquisition of dessication tolerance in seeds; but many LEA proteins are induced by cold, osmotic stress or exogenous abscisic acid, or can even be expressed constitutively [31].

#### Pathogenesis-related genes

PR proteins are induced in response to several pathogen agents (bacteria, viruses and fungi) during the hypersensitive response (HR) and systemic acquired resistance (SAR) [32]. The PR proteins form a heterogeneous family including 17 groups (PR-1 to PR-17) distinguished on the basis of structural homologies [32,33]. However, the biological and biochemical functions of these proteins during the defense reactions and developmental processes are still unclear.

The pathogenesis-related proteins (PR) comprise the vast majority of wine proteins and adversely affect the clarity and stability of wines [34]. The expression of one gene encoding a PR-10 protein was up-regulated during the later stages of grapevine ripening, and especially at the TH+10 stage (Table 4). In general, PR-10 proteins exhibit allergenic, anti-fungal and ribonuclease activities. Robert et al. [35] emphasized the accumulation of PR-10 proteins in grapevine after *Pseudomonas syringae *infection, which was ascribed to HR. Up-regulation of PR-10 expression may be due to attacks of *Botrytis cinerea *which occurred in Champagne vineyards during the last stages of ripening in 2005 and 2006.

In addition, one gene encoding a putative dirigent-like protein (DIR-like) was up-regulated during the berry late ripening (Table 4). This gene displays sequence homology to members of the DIR-b subfamily i.e. PDIR3, PDIR7 and PDIR20 of *Picea glauca *× *engelmanni*, *Picea glauca *and *Picea sitchensis *respectively [36]. The ability of DIR proteins to direct the stereoselective formation of lignans has been previously demonstrated with *in vitro *assays for several members of the DIR-a subfamily from *Forsythia intermedia *[37]. However, the biochemical functions for the members of DIR-b, DIR-c, DIR-d and DIR-e subfamilies are not known so that the members of these subfamilies are referred to as DIR-like. In Sitka spruce trees, the expression of several *DIR *genes was altered by biotic and abiotic stresses, suggesting their implication in plant defense [37].

#### Hormonal control

In the present study, only one gene (FJ822975, termed as *VvCyt1*) encoding a cytokinin histidine-kinase receptor, related to hormone metabolism and regulation of berry development and ripening, was down-regulated throughout the last steps of ripening (Table 4). The *ARABIDOPSIS HISTIDINE KINASE 2 *(*AHK2*) gene is the closest homologue to *VvCyt1*. Cytokinins regulate the development of vascular bundles in inflorescence stems of *Arabidopsis thaliana *via the AHK2 signaling pathway [38]. Cytokinin activity is significant during the early stages of grapevine berry development but decreases later on during ripening [2]. To date, the putative functions of VvCyt1 during the grapevine berry ripening remain to be clarified.

In summary, a total of 12 genes have been shown to be consistently regulated throughout the last steps of the ripening process and can be considered as new indicators of late ripening in Chardonnay. With regard to the five down-regulated genes, an average down-regulation of 2-fold was observed between the TH and TH-7 samples and also between the TH+10 and TH ones. Similarly, an average up-regulation of 3.4-fold and 2.6-fold was observed in the TH versus TH-7 and in TH+10 versus TH samples respectively. These average expression ratios could be related not only to the increase of the sugar to acid ratio (Table 2) throughout the last steps of grapevine ripening but also to the sensory analysis performed (Table 3). The formation of flavors in the ripening grape berry results from the balance of the sugar to acid ratio as well as synthesis of flavor and aromatic compounds [2]. The present study links the sugar to acid ratio, the sensory characteristics and the expression profiles of some specific genes.

### TH versus TH-7 differential gene expression in Chardonnay berries

This comparison allows genes that are differentially expressed just before technological maturity to be identified. Among the genes expressed at the TH-7 and TH stages in WBB and DSB, 52 genes were differentially regulated in TH versus TH-7 berries. In addition to the 12 previously mentioned as up- or down-regulated throughout all the stages of ripening process, 20 more genes associated to a putative function were differentially expressed in TH versus TH-7 WBB and DSB samples (Table 5). Genes representing hypothetical proteins of unknown function are shown in Additional file 1 (Table S1).

**Table 5:** Differentially expressed genes (P < 0.05, ≥1.75-fold) in Chardonnay grapevine berries between theoretical harvest date (TH) and one week before (TH-7) of the 2005 and 2006 growing seasons.

Putative function	Grape Microarray Accession Number (Vv_#)	Grape Nucleotide Accession Number (mRNA)	Grape Gene Accession Number (GSVIVT#)	Most Homologous Arabidopsis Sequence	WBB average ratio	p-value	DSB average ratio	p-value
**Cell wall related genes**								

Alpha-expansin	Vv_10001623	XM_002284822	GSVIVT01007987001	At1g69530	-1.14	0.00032	-0.829	0.00246
Polygalacturonate 4-alpha-galacturonosyltransferase	Vv_10003714	XM_002271124	GSVIVT01020141001	At1g70090	-1.501	0.00027	-1.585	0.00022
Xyloglucan endotransglycosylase/hydrolase	Vv_10011203	XM_002274118	GSVIVT01029170001	At5g57550	-0.884	0.00045	-0.799	0.00062
Xyloglucan endotransglycosylase/hydrolase	Vv_10011021	XM_002274791	GSVIVT01029162001	At5g57560	-1.223	0.00014	-1.2	0.00018
Xyloglucan endotransglycosylase/hydrolase	Vv_10010901	XM_002262725	GSVIVT01031601001	At3g23730	-1.1	0.00033	-1.36	0.00014
Xyloglucan endotransglucosylase/hydrolase	Vv_10011290	XM_002274516	GSVIVT01029166001	At4g25810	-1.472	6.00E-05	-1.475	7.00E-05

**Biotic and abiotic stress related proteins**								

Pathogenesis-related protein 10	Vv_10003874	XM_002274749	GSVIVT01035055001	-	1.542	0.00156	1.086	0.00016
Miraculin-like protein	Vv_10011266	XM_002266394	GSVIVT01012922001	At1g17860	-2.409	9.00E-05	-0.994	0.00104

**Transporters**								

Sulfate transporter	Vv_10001315	XM_002279177	GSVIVT01018028001	At3g51895	-1.207	0.00084	-0.907	0.00169

**Transcription factors**								

TCP transcription factor	Vv_10010249	XM_002272192	GSVIVT01012766001	At1g72010	-1.233	0.00021	-1.038	0.00014
bZIP transcription factor	Vv_10007432	XM_002285275	GSVIVT01014246001	At3g58120	-1.499	7.00E-05	-1.535	5.00E-05

**Miscellaneous**								

Phosphate-induced protein	Vv_10000589	XM_002285726	GSVIVT01009065001	At4g08950	-2.342	0.00021	-1.885	2.00E-04
Phosphate-induced protein	Vv_10000871	XM_002282859	GSVIVT01023873001	At2g17230	-1.141	0.00157	-1.184	0.00098
beta-ketoacyl-CoA synthase	Vv_10004485	XM_002284950	GSVIVT01015472001	At2g26640	-0.845	0.00211	-0.883	0.00212
Metal ion binding protein	Vv_10004892	XM_002281195	GSVIVT01022185001	At4g39700	-1.045	0.00016	-1.325	0.00011
AAA-type ATPase family protein	Vv_10010867	XM_002268820	GSVIVT01023336001	At3g28600	-1.28	0.00026	-1.176	0.00015
AAA-type ATPase family protein	Vv_10012487	XM_002280929	GSVIVT01015385001	At3g24530	-0.805	0.00072	-0.859	0.00099
Aspartyl protease protein	Vv_10002995	XM_002265735	GSVIVT01036694001	At3g12700	-2.0	6.00E-05	-1.975	3.00E-05
Protease inhibitor	Vv_10001691	XM_002266266	GSVIVT01012936001	At1g17860	-3.046	1.00E-05	-0.872	0.00418
PS60 protein/multicopper oxidase	Vv_10000492	XM_002282178	GSVIVT01023902001	At1g76160	-0.922	0.00088	-0.846	0.00141

Genes are organized in functional categories. Ratio values are presented as log2. DSB, densimetrically sorted berries; TH-7, 7-days before theoretical harvest; TH, theoretical harvest; WBB, whole bunch berries.

#### Cell wall-related genes

Fruit development and ripening involve the action of a complex set of enzymes and proteins associated with the disassembly of primary cell wall and reduction in cell-cell adhesion [39]. The expansins, xyloglucan endotransglycosylases/hydrolases and galacturonosyltransferases belong to this set of enzymes.

The expansins are able to plasticize the cellulose-hemicellulose network of plant cell wall. In the literature, three putative *EXP *genes, *Vlexp1*, *Vlexp2*, and *Vlexp3 *have been isolated from Kyoho grape (*Vitis labrusca *x *Vitis vinifera*) berries and their expression was monitored at nine stages of berry development [40]. *Vlexp1 *is the closest homologue to the grapevine *EXPA *gene (GSVIVT01007987001), which is differentially expressed between the TH-7 and TH stages (Table 5). *Vlexp1 *expression increased with berry development up to the half-colored stage and then decreased during the later stages of maturation [40]. In strawberry, *FaEXPA4 *(DQ183068) is the closest homologue of *VvEXPA*. At the protein level, VvEXPA (Table 5) exhibited 79% identity/88% similarity with FaEXPA4. *FaEXP4 *mRNA is strongly expressed throughout fruit development and ripening, and exhibits a slight decrease at the end of maturity in Selva fruits, the firmest cultivar considered in the study of Dotto et al. [41]. This suggests that *VvEXPA *could be associated with the cell expansion and grapevine berry ripening (Table 5).

Changes in the pectin matrix are regarded as an important factor that affects the cell wall structure during the fruit ripening and senescence [42]. α-(1,4)-Galacturonosyltransferases catalyze the addition of (1,4)-linked α-D-galacturonosyl residues onto the nonreducing end of homogalacturonan chains [43]. One gene encoding such a putative galacturonosyltransferase was down-regulated at the TH stage in comparison with the TH-7 one.

Xyloglucan is the principal hemicellulose component in the primary cell walls of non-graminaceous plants, and accounts for 10% of the cell wall composition in grapevine berries [44]. During the fruit ripening process, xyloglucan degradation is the terminal cell wall degradation that occurs [45]. Xyloglucan endotransglycosylases/hydrolases (XTH) are involved in splitting and/or reconnecting xyloglucan cross-links in a new position, and their action helps satisfy the contradictory needs of growing and/or differentiating tissues [46]. Nunan et al. [47], Deluc et al. [7] and Glissant et al. [48] have already reported the involvement of a few XTH genes during the grapevine berry development. However, none of them corresponds to the four XTH (XM_002274118, XM_002274791, XM_002262725 and XM_002274516), which are down-regulated between TH-7 and TH stages (Table 5). The four XTH are closely related to the tomato *LeXTH3 *or *SiXTH3 *[49] (XM_002274118, XM_002274791), litchi *LcXET3 *[50] (XM_002262725) and Charentais melon *CmXTH3 *[51] (XM_002274516) genes respectively. The expression profiles of these genes suggest their involvement in the depolymerization of xyloglucan fraction in relation to fruit softening.

While the enzymatic basis of this process has not been established, cell wall-modifying proteins have been suggested to play a synergistic role in the restructuring of the cellulose-xyloglucan-pectin network during the fruit ripening [45].

#### Plant defense proteins

Another PR-10 gene is up-regulated during the grapevine ripening, especially between TH-7 and TH stages. Among the stress-related genes, one gene homologous to a miraculin-like protein is also down-regulated (Table 5). A miraculin is a plant protein purified from extracts of "miracle fruit" berries (*Synsepalum dulcificum*) which is able to modify a sour taste into a sweet taste [52]. In Citrus and *Poncirus trifoliata*, a *miraculin *homologue is down-regulated by cold stress (which reduces water availability) [53]. In coffee, *Coffea *miraculin (*CoMir*) expression was prominent during the early stages of fruit development and then repressed throughout fruit maturation [54]. Like the up-regulation of VvGolS (see above), the down-regulation of the grapevine *miraculin-like *gene may be a response to a decrease of water availability.

#### Transporter protein

A large number of genes encoding proteins with functions in the transport of water, ions, sugars, and non-specific substrates show differential expression during berry ripening [7,55,56].

Among these compounds, inorganic sulfate is acquired from the soil as a major source of sulfur nutrient in higher plants. The long distance transport of sulfur is in part mediated by phloem translocation of sulfate or sulfur-containing metabolites, such as glutathione and S-methyl-Met [57]. A member of the group 3 sulfate transporters (XM_002279177) is down-regulated at the TH stage compared to TH-7 (Table 5). The expression of group 3 sulfate transporters is not affected by the sulfate status of the plant [58]. The role of such a transporter in grapevine berry ripening is still unknown although three others group 3 sulfate transporters were already identified as differentially expressed in tissues of grapevine berry [55].

#### Transcription factors

Two genes encoding a basic leucine zipper (bZIP) and a TCP transcription factors exhibited lower expression at the TH stage compared with the TH-7 one (Table 5). Although the Teosinte Branched1, Cycloidea and PCF (TCP) domain protein families, which belong to the family of bHLH-type transcription factors, are thought to be key regulators of morphological traits [59], no data are available about the involvement of such a transcription factor in the regulation of fruit ripening.

### TH+10 versus TH differential gene expression in Chardonnay berries

Among the genes expressed at the TH+10 and TH stages in WBB and DSB, 53 genes were differentially expressed in TH+10 versus TH berries. In addition to the 12 previously mentioned as up- or down-regulated during all the end of the ripening process, 24 other genes associated to a putative function were differentially expressed in TH+10 versus TH WBB and DSB samples (Table 6). Among the 53 genes differentially expressed in TH+10 versus TH berries, some of them did not have any known function and they are shown in Additional file 2 (Table S2).

**Table 6:** Differentially expressed genes (P < 0.05, ≥1.75-fold) in Chardonnay grapevine berries between 10-days after theoretical harvest (TH+10) and theoretical harvest date (TH) of the 2005 and 2006 growing seasons.

Putative function	Grape Microarray Accession Number (Vv_#)	Grape Nucleotide Accession Number (mRNA)	Grape Gene Accession Number (GSVIVT#)	Most Homologous *Arabidopsis *Sequence	WBB average ratio	p-value	DSB average ratio	p-value
**Aroma related genes**								

Valencene synthase (VvValCS)	Vv_10004183	FJ696653/AY561843	GSVIVT01036322001	At5g23960	2.031	2.00E-05	1.823	2.00E-05

**Pathogenesis-related genes**								

Pathogenesis-related protein 1 (PR-1)	Vv_10011243	AJ536326	GSVIVT01037015001	At2g14580	2.049	3.00E-05	1.558	0.00024
Pathogenesis-related protein 1 (PR-1)	Vv_10004981	XM_002274239	GSVIVT01037014001	At2g14610	2.059	0.00016	1.637	0.00029
Beta-1,3-glucanase (PR-2)	Vv_10010418	AF239617	GSVIVT01035013001	At4g16260	1.566	0.00014	0.994	0.00133
Thaumatin-like protein (PR-5)	Vv_10000872	XM_002282994	GSVIVT01019840001	At4g11650	1.337	0.00018	1.273	0.00019
Leucine-rich repeat protein	Vv_10000077	XM_002263817	GSVIVT01032059001	At3g20820	-1.26	0.00015	-1.668	0.0012

**Stress-related genes**								

Heat shock protein	Vv_10011030	XM_002281184	GSVIVT01016426001	At5g59720	-1.157	0.00016	-1.399	0.00027
Heat shock protein	Vv_10000006	XM_002281358	GSVIVT01016429001	At3g46230	-0.951	0.00788	-1.133	0.00038
Heat shock protein	Vv_10011029	XM_002281318	GSVIVT01016428001	At3g46230	-1.136	7.00E-04	-1.351	0.00013

**Growth and development-related genes**								

TFL1C protein	Vv_10003390	XM_002278819	GSVIVT01010598001	At5g62040	0.929	0.00023	1.208	0.00025
Rapid ALkalinization Factor-like protein	Vv_10004862	XM_002282632	GSVIVT01022118001	-	-0.962	0.00191	-1.067	0.00235

**Cell wall-modifying enzymes**								

Polygalacturonase-like protein	Vv_10013430	XM_002278894	GSVIVT01019405001	At4g23500	1.376	0.00066	1.335	0.00031
Pectate lyase (VvPL1)	Vv_10010773	AY043234	GSVIVT01029048001	At1g04680	-1.014	0.00031	-1.677	0.00043

**Hormone metabolism and regulation**								

Auxin-responsive protein	Vv_10002615	XM_002284085	GSVIVT01018099001	At5g43700	1.08	0.00032	0.946	0.00047
Auxin-responsive protein	Vv_10009542	XM_002279919	GSVIVT01021779001	At1g04240	1.736	7.00E-05	1.415	0.00045
Gibberellin 2-oxidase	Vv_10009047	XM_002268137	GSVIVT01012628001	At4g21200	-1.197	0.00069	-1.046	0.00037

**Transporters and trafficking**								

Aquaporin (TIP1;2)	Vv_10003817	DQ834702	GSVIVT01033677001	At2g36830	-0.802	0.00097	-1.286	0.00022
Vacuolar pyrophosphatase								
(vpp2)	Vv_10000514	AJ557256	GSVIVT01012841001	At1g15690	-0.997	0.00018	-0.911	0.00048

**Miscellaneous**								

Homeobox leucine zipper protein	Vv_10004955	XM_002262914	GSVIVT01027407001	At3g61890	-1.065	0.00029	-1.076	3.00E-04
Heavy-metal-associated domain-containing protein	Vv_10002809	XM_002277832	GSVIVT01025598001	At5g02600	-0.975	0.00029	-0.95	0.00046
GDSL-motif lipase/hydrolase family protein	Vv_10000511	XM_002271815	GSVIVT01030528001	At4g26790	-0.82	0.00068	-0.853	0.00087
Amino acid transporter	Vv_10014047	XM_002283432	GSVIVT01011401001	At3g28960	-1.053	0.00122	-1.186	0.00031
Copper ion binding oxidoreductase	Vv_10001170	XM_002275642	GSVIVT01037479001	At5g21105	-0.849	0.00036	-0.866	0.00052
Peptidase/subtilase	Vv_10008612	XM_002278256	GSVIVT01015069001	At2g05920	-1.134	0.00068	-1.101	0.00131

Genes are organized in functional categories. Ratio values are presented as log2. DSB, densimetrically sorted berries; TH, theoretical harvest; TH+10, 10-days after harvest; WBB, whole bunch berries.

#### Aroma and flavor related genes

**Figure 4:**
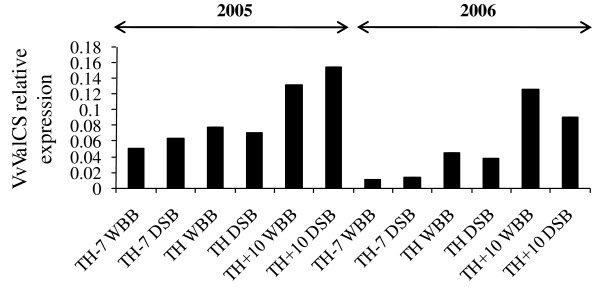
**Bar diagram of *Vitis vinifera (+)-valencene synthase *(*VvValCS*) transcript abundance: a comparison of qRT-PCR data of Chardonnay whole bunch and densimetrically sorted berries harvested at three ripening stages in 2005 and 2006**. The mRNA level was expressed relative to controls (set at 1), reference gene *EF1-α*. RT-PCR data are reported as means ± SE (error bars) of n = 3 technical replicates. DSB, densimetrically sorted berries; TH-7, 7-days before theoretical harvest; TH, theoretical harvest; TH+10, 10-days after harvest; WBB, whole bunch berries.

Several flavor and aroma compounds, such as pyrazines, terpenes or shikimic acid derivatives, are responsible for the character of wines and contribute to their quality [60,61]. Among them, the terpenoid volatiles which derive from isoprene units are crucial for the fruity and floral aromas and flavors of wine. Furthermore, the higher terpenes may also be responsible for the diesel or fuel off-flavors of wines. During Chardonnay berry ripening, the transcript abundance of the (+)-valencene synthase (*VvValCS*; FJ696653/AY561843) gene, encoding an enzyme involved in sesquiterpene biosynthesis, increased significantly in the TH+10 berries compared with the TH samples (WBB and DSB) (Table 6). The *VvValCS *expression pattern was validated by qRT-PCR (Figure 4). The increased transcript abundance of the *VvValCS *gene is likely an indicator for the synthesis of some aroma-related compounds at the latest stages of the ripening process. Deluc et al. [62] investigated the expression profile of the *VvValCS *gene during grapevine berry development under normal and water stress culture conditions in Chardonnay and Cabernet Sauvignon cultivars. At the end of ripening, the *VvValCS *transcript profile found in Chardonnay was similar to our result. Lücker et al. [63] also demonstrated the importance of *VvValCS *transcript in the production of terpenoid compounds during the late ripening stages of Gewürztraminer cultivar. Thus, VvValCS may play a key role in flavor and aroma volatile production of at least two aromatic white grapevine cultivars; and this independently of climatic conditions and vintages.

#### Pathogenesis-related genes

Five genes related to pathogen attack responses were identified as differentially expressed between TH+10 and TH phases (Table 6). Among them, the genes encoding two PR-1 (AJ536326, XM_002274239), a *b*-1,3-glucanase (PR-2; AF239617) and a thaumatin-like protein (PR-5; XM_002282994) displayed similar transcript profiles and were up-regulated at the TH+10 stage (versus the TH period). A putative *leucine-rich repeat *(*LRR*; XM_002263817) gene was down-regulated.

In grapevine berry, some PR genes are expressed at a constitutive level throughout berry development whereas others are only induced at the véraison stage, for example some of grape ripening-induced proteins (GRIP) [64]. Among the PR proteins, the subfamily PR-1 is comprised of low-molecular-weight proteins of unknown biochemical function, but may be involved in the response to environmental stresses [32].

The transcript level of a *b*-1,3-glucanase (AF239617) was up-regulated at the TH+10 stage compared with the TH samples. The *b*-1,3-glucanases represent one of the most investigated families of PR proteins in grapevine [55,65,66]. Thus, isozymes of glycosyl hydrolase family 17 hydrolyze *b*-1,3-glucan polysaccharides found in the cell wall matrix of plants and fungi, enabling these plant enzymes to fulfill diverse biological functions in plant defense and plant development. In grapevine, the *b*-1,3-glucanases are usually associated with the response and defense to pathogen attacks. According to Roy Choudhury et al. [67], *b-*1,3-glucanases also play a role in fruit ripening and/or softening. During grape berry development and in post-harvest, the presence of abundant active PR proteins in Cabernet Sauvignon berry skins, especially *b*-1,3-glucanases, is not sufficient to protect berries from pathogen infection [66]. Moreover, the abundance of *b*-1,3-glucanase proteins in the berry proteome is not well correlated with enzymatic activity [66]. In the present experiments, although several berries of harvested bunches were damaged by pathogens, especially by *Botrytis cinerea*, all berries selected for RNA extractions were healthy. It is not possible to conclude unequivocally on whether this *b-*1,3-glucanase plays a role during the late stage of fruit ripening or in defense against pathogens.

In grapevine, several thaumatin-like or osmotin-like proteins (PR-5 proteins) were identified [68,69] and their powerful anti-fungal activity was established *in vitro*. The thaumatin-like protein (XM_002282994) up-regulated at the TH+10 stage may be involved in antifungal response and/or to osmotic adjustment.

In plants, the LRR proteins mediate protein-protein interaction and participate in many biologically important processes, such as hormone-receptor interactions, trafficking, plant development or organ differentiation [70]. Furthermore, the involvement of LRR proteins is essential in plant defense and resistance to diseases or pathogen attacks [70,71]. It can be hypothesized that genes encoding LRR proteins are induced or up-regulated at the onset of pathogen infections. LRR proteins may play a role in the signal transduction cascades which up-regulate PR genes. The down-regulation of one LRR, observed at the TH+10 stage versus the TH one, could be related to a negative feedback.

#### Stress-related genes

The expression of three genes encoding heat shock proteins (HSP) (XM_002281184, XM_002281358, XM_002281318) is down-regulated at the TH+10 stage (Table 6). They are members of class I smHSPs (small HSP) and share 95% identity. In plants, the smHSPs are induced upon stress and plant tolerance to stress, including drought, salinity or low temperatures [reviewed in 72]. It was suggested that besides their function during the stress response, smHSPs are involved in specific biological processes of plant development. In addition to protecting photosystem II from a temperature-dependent oxidative stress, the tomato smHSP21 also promotes color changes during fruit maturation [73]. The three grapevine *smHSP *down-regulated during the last phase of maturation are closely related to the strawberry *smHSP njjs4 *(U63631) gene [74], whose transcripts are accumulated in fruits (receptacle), but not in roots, flowers and leaves [74]. The *njjs4 *gene expression is not only organ-specific but also stage-specific. Its expression profile suggests that njjs4 smHSP plays an important function in fruit development, especially during the early fruit ripening process. A similar role can be hypothesized for the grapevine berry *smHSP *XM_002281184, XM_002281358 and XM_002281318.

#### Growth and development-related genes

Among the genes with up-regulated expression between TH+10 and TH stages, the presence of the *VvTFL1C *and *Rapid ALkalinization Factor-like *(*RALF-like*) genes, potentially involved in plant development, can be highlighted (Table 6).

The *FLOWERING LOCUS T/TERMINAL FLOWER 1 *(*FT/TFL1*) gene family encodes proteins with similarity to phosphatidylethanolamine binding proteins which function as flowering promoters and repressors [75]. The FT and TFL1 proteins display opposite functional roles. *VvFT *transcript is mainly expressed in inflorescences and berries; its role in promoting flowering has been demonstrated, but its role in fruit development remains unclear [76]. Carmona et al. [75] have investigated the gene expression patterns of *FT/TFL1 *gene family in grapevine. The *VvTFL1C *transcript level is in agreement with a role of this gene in vegetative development and maintenance of meristem indetermination. Moreover, *VvTFL1C *mRNA is weakly detected during the phase III of berry development corresponding to the maturation period. However, the role and especially the up-regulation of this gene at the TH+10 stage remains to be understood.

The Rapid ALkalinization Factor (RALF) proteins are small peptides which were initially associated with plant wound or defense responses. However, recent studies show the inability of *RALF *genes to be induced by pathogens or stress elicitors and suggest that RALF could play other roles *in planta *[77]. The characterization of *RALF-like *genes from *Solanum chacoense *supports the view of a developmental role for this multigenic family in plants [77]. The *ScRALF3 *gene from *Solanum chacoense *is the most homologous sequence to the grapevine *RALF-like *gene (XM_002282632). The *ScRALF3 *gene appeared to be expressed almost exclusively in ovary tissues and fruits where its transcripts became less abundant during fruit maturation. The expression profile of *ScRALF3 *is consistent with the grapevine *RALF-like *one.

#### Cell wall-modifying enzymes

Cell wall disassembly and modifications to the pectin fraction are some of the most apparent changes that occur in the cell wall during the ripening process [78]. During grapevine berry ripening, the progressive depolymerisation of cell wall pectin structure occurs through the action of polysaccharide hydrolases including polygalacturonases (PG). PG, an important pectolytic glycanase, is the primary enzyme playing a significant role in pectin dissolution *in vivo*. In the skin of developing grapevine berries, *VvPG1 *transcripts levels correlate with berry softening, and *VvPG1 *and *VvPG2 *transcript levels increase during the skin ripening [42]. Table 6 reveals shows that a gene (XM_002278894) encoding a putative PG-like protein is up-regulated. To date, data available about the contribution of PG-like proteins during the last steps of fruit ripening process are scarce.

Among the cell wall-modifying enzymes, the pectate lyases (PL, EC 4.2.2.2) also possess a pectinolytic activity. They catalyze the eliminative cleavage of de-esterified pectin and generate oligosaccharides with unsaturated galacturonosyl residues.

Various studies related to biochemical and physiological changes occurring during the softening and ripening of climacteric (mango [79]) and non-climacteric fruits (grape [47], strawberry [80]), suspected a role for pectate lyases in pectin degradation throughout pulp softening and fruit ripening. During Chardonnay ripening, the grapevine *VvPL1 *gene (AY043234) is significantly down-regulated at the TH+10 stage compared to the harvest phase. Nunan et al. [47] previously showed a similar expression pattern in Muscat Gordo Blanco ripening berries. A high level of *VvPL1 *mRNA was present during the maturation process, particularly at the véraison, followed by a progressive decrease of *VvPL1 *transcript level until over-maturation phase [47]. Furthermore, the *VvPL1 *gene is homologous to the mango *MiPel *(AY987389) [79] and strawberry *plC *(AF339025) [80] genes. The onset of mango softening and ripening is closely related to an increase in the *MiPel *PL gene expression, PL activity and pectin solubilization [79]. Taken together, these data suggest a crucial role of *VvPL1 *gene during the berry ripening of white grapevine cultivars.

#### Hormone metabolism and regulation

Among the hormone-related genes, only two genes associated with auxin and gibberellic acid metabolism and signaling were differentially expressed between the TH+10 and TH harvest stages, i.e. an *auxin-responsive *gene and a *gibberellin 2-oxidase 1 *gene (Table 6).

Auxins are known to mediate the onset of berry development in grapevine [2,81]. Moreover, indole-3-acetic acid (IAA) content reaches its maximal level just after anthesis and then declines to very low levels in the ripe fruit [2,82]. Two *auxin-responsive *genes (XM_002284085, XM_002279919) homologous to the cotton *Gbiaa-re*, which is a member of plant *AUX/IAA *gene family [83] were identified. It can be hypothesized that these grapevine auxin-response proteins act as activators or repressors of genes mediating the various auxin responses. In cotton, Gbiaa-re exhibits conserved integrated domains of the "AUX_IAA, AUX/IAA family" and the expression of *Gbiaa-re *gene is inducible by IAA [83]. Yang et al. [84] highlighted the putative function of this gene, which was considered as a transcription factor, during cell wall regeneration in cotton protoplasts. Surprisingly, the two grapevine *auxin-responsive *genes are up-regulated at the TH+10 stage compared with the TH stage, whereas IAA content is very low at the end of berry ripening [82].

One *GA 2-oxidase *gene involved in GA biosynthesis is down-regulated in berries harvested at the TH+10 stage compared with the berries harvested 10 days before (Table 6). In the literature, it was hypothesized that the GA 2-oxidase oxidizes the precursors of bioactive GAs and plays a key role in determining or regulating the amounts of active GAs in plants [85]. The characterization of a grapevine gibberellic acid (GA) dwarf mutant, provided genetic evidence that GAs inhibit the flowering in grapevine [86]. However, its function in berry ripening remains to be tested.

#### Transporters and trafficking

Two distinct primary proton pumps, the H+-transporting adenosine triphosphatase (V-ATPase) and H+-translocating inorganic pyrophosphatase (V-PPase) are localized in the plant vacuolar membrane. Their activity creates a proton electromotive force allowing the secondary active transport of inorganic ions, sugars and organic acids. In grape, Terrier et al. [87] and Venter et al. [88] already identified and characterized two isoforms of the *V-PPase *gene named *VVPP1 *and *vpp2 *respectively. During the grapevine berry ripening, the V-PPase activity apparently increases in parallel with the transcript levels of *vpp2 *and *VVPP1 *[87,88]. The expression pattern of *Vpp2 *is also modulated by abiotic stresses such as cold [88]. In the present study, the transcript level of *vpp2 *gene is down-regulated in Chardonnay cultivar at the TH+10 stage in comparison with the theoretical harvest stage (TH) (Table 6). This result could be in agreement with a potential reduced V-PPase activity 10-days after theoretical harvest date (TH+10).

In our experiments, a similar expression pattern was shown for the aquaporin *TIP1;2 *(DQ834702) gene, encoding a water channel protein. This confirms and extends earlier data showing that the expression of *VvTIP1;2 *is down-regulated during Cabernet Sauvignon berry ripening [56]. As suggested by Tyerman et al. [89], aquaporins might also play a role in the regulation of berry hydraulic conductance, especially between véraison and harvest when a drastic reduction of berry hydraulic conductance occurs.

In summary, the pattern of all the genes differentially expressed between the TH and TH-7 stages and between the TH+10 and TH stages can be considered as an indicator of the optimal harvest date for the Chardonnay cultivar. Taken together, these genes constitute a set of potential ripening indicators distinguishing the optimal harvest date from under-maturation and over-maturation phases.

It is noteworthy that among the differentially expressed genes, only two transcription factors (a bZIP (XM_002285275) and a TCP (XM_002272192), Table 5) were found, and were down-regulated between the TH and TH-7 stages. The other genes encoded for enzymes or structural proteins. This suggests that no major reprogramming of transcription patterns occurs at the end of the ripening.

Unfortunately, these expression profiles cannot be compared and validated to the other large-scale expression profiling studies performed to analyze transcription changes during berry development and ripening [6-8]. Indeed, the TH-7, TH and TH+10 steps of berry ripening have never been used together to investigate the last stages of grapevine ripening. Moreover, the intervals used here between two sampling times (seven to ten days) are much shorter than those used in previous studies [6-8].

### Is there an indicator of ripening status spreading during Chardonnay berry ripening?

At the TH stage in comparison with the TH-7 stage, the transcript level of the gene encoding a putative S-adenosyl-L-methionine:salicylic acid carboxyl methyltransferase (VvSAMT, XM_002262640) is down-regulated in DSB corresponding to the most representative density of a given harvest date (Table 7). The same gene is down-regulated only at the TH+10 stage in comparison with the theoretical harvest stage (TH) in WBB (Table 7). Down-regulation of *VvSAMT *is thus detected earlier in DSB than in WBB. It can be hypothesized that this gene is an early indicator forecasting bunch ripening. A SAMT enzyme is responsible for the formation of methyl salicylate which is part of secondary metabolites and especially of volatile methyl esters [90]. Methyl salicylate belongs to plant fragrant compounds and contributes to floral scent and flavor ingredients found in fruits. Methyl salicylate, and as a consequence SAMT are also thought to play a role in inter- and intraplant communications during the plant defense against pathogen infections. These functions of the SAMT multigenic family were assigned following detailed biochemical testing [90,91]. Our data characterize the first association of a *SAMT *gene with the last phase of berry ripening. In Chardonnay, the specific expression pattern observed may be more related to a slowing down of flavor compound synthesis/accumulation than an involvement of this *VvSAMT *gene in response to biotic stress. Although some bunches suffered pathogen attacks in the vineyard at the TH+10, the samples used for microarray analysis were selected free of pathogens.

**Table 7:** *VvSAMT *gene expression (P < 0.05, ≥1.75-fold) in Chardonnay grapevine berries all along the investigated ripening periods of the 2005 and 2006 growing seasons.

Putative function	Grape Microarray Accession Number (Vv_#)	Grape Nucleotide Accession Number (mRNA)	Grape Gene Accession Number (GSVIVT#)	Most Homologous *Arabidopsis *Sequence	WBB average ratio (TH vs TH-7)	p-value	WBB average ratio (TH+10 vs TH)	p-value	DSB average ratio (TH vs TH-7)	p-value	DSB average ratio (TH+10 vs TH)	p-value
S-adenosyl-L-methionine:salicylic acid carboxyl methyltransferase (VvSAMT)	Vv_10000965	XM_002262640	GSVIVT00024874001	At3g21950	NS	NS	-0.899	0.00325	-0.932	0.00223	NS	NS

Ratio values are presented as log2. DSB, densimetrically sorted berries; NS, non significant values; TH-7, 7-days before theoretical harvest; TH, theoretical harvest; TH+10, 10-days after harvest; WBB, whole bunch berries.

### Validation of some Chardonnay ripening indicators within another cultivar

It is interesting and important to determine whether the genes which are consistently affected during the late stages of Chardonnay (white cultivar) ripening in Champagne vineyard (France) are also affected for a red variety grown under completely different conditions. To this end, the expression profiles of those genes were studied in Cabernet Sauvignon (red cultivar) grown in control chambers. In controlled rooms, Cabernet Sauvignon rooted cuttings were subjected to two kinds of temperature regimes either 30°C days and 25°C nights (high temperature regime) or 20°C days and 15°C nights (low temperature regime). Three parameters of the progression of berry development and ripening i.e. berry volume, percentage of colored berries and total soluble solids (°BRIX) were investigated.

**Figure 5:**
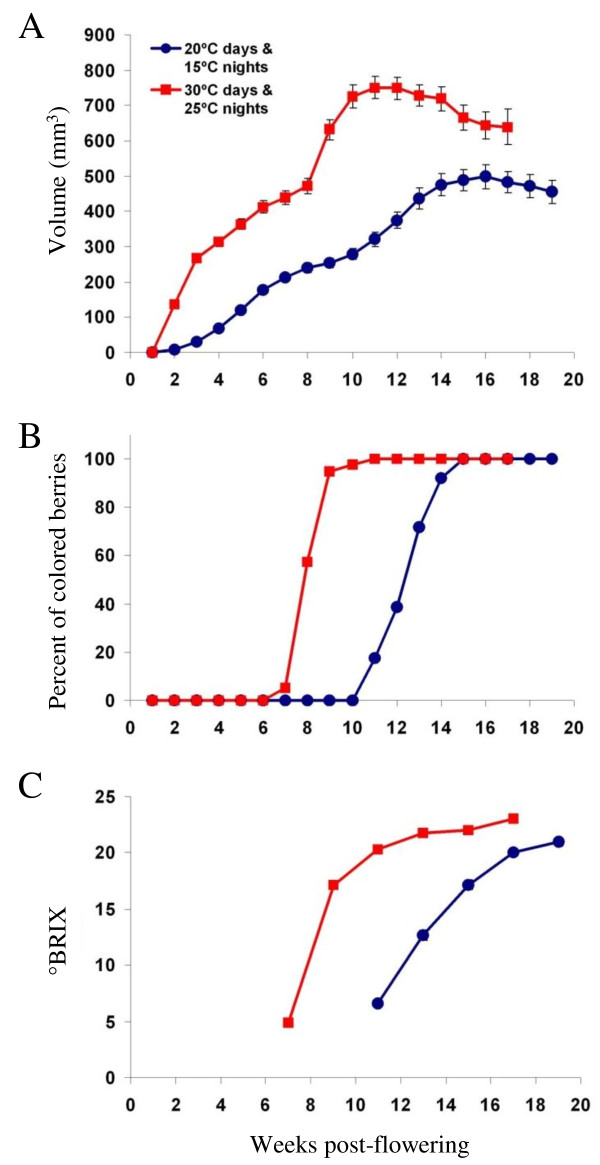
**Progression of Cabernet Sauvignon berry development and ripening**. Changes of various parameters i.e. the berry volume (A), percentage of colored berries (B) and total soluble solids (°BRIX) in the berry juice (C) were investigated in controlled rooms. Cabernet Sauvignon rooted cuttings were subjected to either 30°C days and 25°C nights or 20°C days and 15°C nights temperature regimes. Data are reported as means ± SE (error bars).

The volume of Cabernet Sauvignon berries followed a typical double sigmoidal growth curve, characteristic of the grape berry development [1], whatever the temperature regime (Figure 5A). However, results observed for berry volume, percentage of colored berries and total soluble solids indicated a precocity of véraison and maturation with high temperature regime instead of low temperature one.

The volume of berries subjected to the high temperature regime (Figure 5A) increased during the first 6 weeks of development to approximately 450 mm^3^, followed by a lag phase in the berry expansion until 9 weeks post-flowering, after which the volume began to increase again. The volume of this kind of berries peaked at week 11 (approximately 750 mm^3^) and then decreased to a final value of 650 mm^3 ^at harvest. The berries subjected to 20°C days and 15°C nights began the véraison lag phase more than two weeks after berries subjected to 30°C days and 25°C nights and their berry volume peaked at 16 weeks post-flowering to approximately 500 mm^3^. So an increase of 10°C during days and nights influences not only the precocity of véraison and maturation but also berry growth.

The onset of ripening in red grapes is indicated by an increase in softness, sugar content, berry size, and also by the development of skin color. In the current experiments, the coloration of berries exposed to high temperature regime began four weeks (7 weeks post-flowering) before the berries subjected to 20°C days and 15°C nights (11 weeks post-flowering) (Figure 5B). For high temperature berries, only two weeks were necessary to obtain 95 percent colored berries instead of four weeks for low temperature Cabernet Sauvignon berries.

Similarly, total soluble solids (measured as °BRIX) of high temperature berries began to increase 8 weeks post-flowering and continued to increase, reaching a value of 24°BRIX 17 weeks post-flowering (Figure 5C). As previously evidenced for the berry volume and percentage of colored berries, the total soluble solids of berries exposed to the low temperature regime only began to increase four weeks after the berries grown under the high temperature regime and attained a maximal value of 22°BRIX 19 weeks post-flowering (Figure 5C). From the data, véraison is considered to occur between 7 and 8 weeks post-flowering for berries subjected to 30°C days and 25°C nights, and between 10 and 11 weeks post-flowering for berries exposed to 20°C days and 15°C nights.

Taken together the combination of these observations implies that a higher temperature substantially hastened berry development, ripening and consequently maturation. This is in general agreement with existing knowledge on the influence of temperature on grapevine berry development especially on ripening process [92].

Gene expression analysis was performed on Cabernet Sauvignon berries harvested from 7 to 19 weeks post-flowering i.e. from véraison of 30°C days and 25°C nights berries to harvest of berries exposed to 20°C days and 15°C nights. The expression profiles of nine candidate genes of Chardonnay late ripening were quantified and analyzed: *VvCCD4a*, *VvPAL2*, *VvGolS*, *VvLEA1*, *VvDIR-like*, *VvHKR*, *miraculin-like *gene (XM_002266394), *VvValCS *and *VvSAMT*. Among them, four genes, *VvCCD4a*, *VvPAL2 *and *VvDIR-like*, were up-regulated throughout the last phases of Chardonnay ripening (TH-7, TH, TH+10). Similarly *VvGolS*, *VvLEA1 *and *VvHKR *were down-regulated throughout the same last phases of Chardonnay ripening. The *miraculin-like *gene was down-regulated in TH versus TH-7 berries and *VvValCS *was a member of the 53 genes differentially regulated in TH+10 versus TH berries.

First of all the general aspects of expression profiles of seven of the nine genes investigated are similar in all culture conditions investigated, vineyard or controlled environment rooms, and whatever grapevine cultivars considered, Chardonnay (white cultivar) or Cabernet Sauvignon (red cultivar).

**Figure 6:**
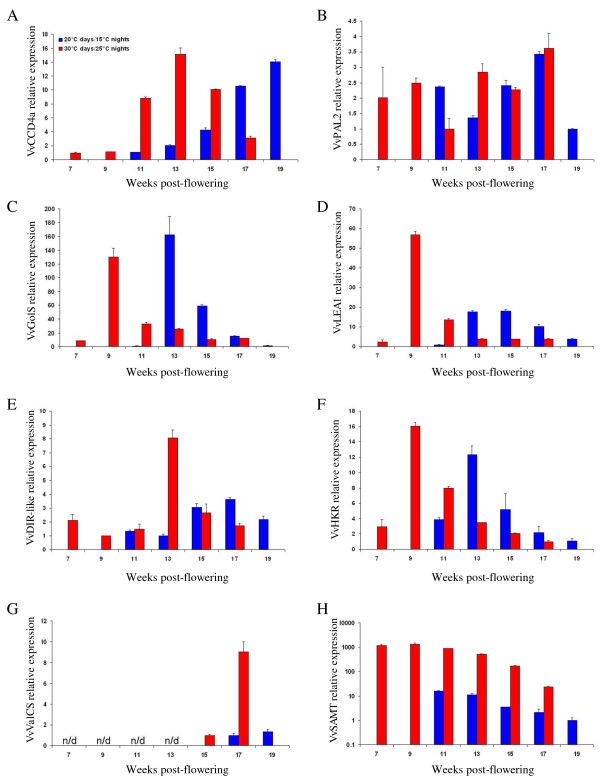
**Transcript abundances of eight potential ripening indicators in *Vitis vinifera *cv. Cabernet Sauvignon**. The expression profiles of *VvCCD4a *(A), *VvPAL2 *(B), *VvGolS *(C), *VvLEA1 *(D), *VvDIR-like *(E), *VvHKR *(F), *VvValCS *(G) and *VvSAMT *(H) were investigated from véraison to harvest. Berries were subjected to either 30°C days and 25°C nights or 20°C days and 15°C nights temperature regimes. The mRNA level was expressed relative to the lowest level of expression detected in any sample for each gene, reference genes *Ubiquitin*, *Actin *and *EF1-α*. RT-PCR data are reported as means ± SE (error bars) of n = 3 technical replicates.

Throughout the late ripening phase of Cabernet Sauvignon berries, *VvCCD4a *and *VvDIR-like *expression profiles evolved differently depending on the temperature regime (Figures 6A and 6E). A 20°C days and 15°C nights regime induced an up-regulation of *VvCCD4a *and *VvDIR-like *from véraison to harvest (as evidenced above during the last stages of Chardonnay ripening). Conversely, a 30°C days and 25°C nights regime generated an up-regulation of *VvCCD4a *and *VvDIR-like *from véraison until 13 weeks post-flowering, equivalent to 22°BRIX, and then a down-regulation until harvest.

The expression profiles of *VvGolS, VvLEA1 *and *VvHKR *during ripening phase is similar between Chardonnay berries harvested from a vineyard and Cabernet Sauvignon berries subjected to a 20°C days and 15°C nights temperature regime (Table 4 and Figures 6C, 6D and 6F). In both experiments, a down-regulation of *VvGolS*, *VvLEA1 *and *VvHKR *expression profiles was evidenced. A down-regulation of *VvGolS*, *VvLEA1 *and *VvHKR *was also shown in Cabernet Sauvignon berries exposed to 30°C days and 25°C nights from 9 to 17 weeks post-flowering (Figures 6C, 6D and 6F).

Among the nine genes investigated, the *VvPAL2 *differs from the others because its expression profile showed no similar variation throughout the last steps of Chardonnay and Cabernet Sauvignon ripening (Table 4 and Figure 6B). *VvPAL2 *may not behave the same between Cabernet Sauvignon and Chardonnay berries as it may be influenced by anthocyanin production in the Cabernet Sauvignon berries compared to the Chardonnay ones.

Similarly, the *miraculin-like *gene can be distinguished from the others because throughout ripening the expression profile of this gene is completely the opposite between Chardonnay and Cabernet Sauvignon berries (data not shown). This gene behaves differently depending on the cultivar and may only be viewed as a cultivar-specific indicator of ripening as *VvPAL2*.

In Cabernet Sauvignon, *VvValCS *was only expressed during the last two weeks of the ripening phase (Figure 6G) and an up-regulation was noticed in all temperature regimes investigated (as evidenced in Chardonnay samples).

Similarly, the *VvSAMT *gene was down-regulated throughout the maturation phase of the grapevine berry development from véraison to harvest date, in all temperature regimes investigated, and particularly during the last steps of ripening (Figure 6H).

To conclude, seven ripening indicators i.e. *VvCCD4a*, *VvGolS*, *VvLEA1*, *VvDIR-like*, *VvHKR*, *VvValCS *and *VvSAMT *evidenced from the experiments performed on Chardonnay berries could also be considered as late ripening indicators of Cabernet Sauvignon, and were thus validated for a major white and a major red grapevine cultivar. Some of them have their expression profiles influenced by temperature such as *VvCCD4a, VvDIR-like *and to a lesser extent *VvLEA1 *while the temperature regime has no impact on *VvGolS*, *VvHKR*, *VvValCS *and *VvSAMT *expression profiles even if precocity of fruit maturation was noticed with berries exposed to the higher temperature regime.

The trends of Chardonnay and Cabernet Sauvignon are similar despite their different growth conditions and their different genetic background and despite the fact that the timing of the sampling is somewhat different between the sample sets. This shows the robustness of the gene expression patterns. The Cabernet Sauvignon series grown at the higher temperature reached a more mature final stage of development than the other series but this does not invalidate the trends observed for the earlier, and comparable, period of development.

In the context of climatic changes which alters grapevine physiology, berry growth ripening and content, the identification of genes linked to the late stages of grapevine ripening is important.

## Conclusions

The last steps of grapevine ripening involve a correlative differential expression of numerous genes. However, based on the comparison of whole bunches vs densimetrically sorted berries, of two vintages, and of two cultivars (white and red) grown either in vineyard or greenhouse conditions, only a limited set of the tested genes (*VvCCD4a*, *VvGolS*, *VvLEA1*, *VvDIR-like*, *VvHKR*, *VvValCS *or *VvSAMT) *showed a consistent expression pattern. They might be used directly or indirectly as potential indicators of adequate ripening for optimal wine quality.

Direct use of gene expression profiling is already used commercially to monitor pear and apple ripening, and the physiological status of ornamental and forest species (http://www.nsure.eu). Indirect assays of the proteins coded by these genes by specific antisera, or of the metabolites synthesized by these proteins (CCD4, GolS, ValCS, SAMT) may also be envisaged. Each of these methods has potential technical limitations. For example, the antisera must be specific, sensitive, and the protein targeted must be abundant enough. Metabolite assays rely on the assumption that the enzymes identified above are the only ones limiting their synthesis. Therefore, further work is needed to investigate these different possibilities. In order to reach a more precise idea of the ripening status, it may also be useful to combine a ratio or difference of activities of both up- and down-regulated genes/proteins/metabolites rather than to rely only on up- or down-regulated genes. Moreover, the precise ratio or set of indicators determined for optimum maturity will vary with the maturity and the style of wine that the wine maker wishes to make.

## Methods

### Plant material

#### - Samples from vineyard

Experimental material was harvested during the 2005 and 2006 growing seasons from *Vitis vinifera *L. cv. Chardonnay grapevines, grown at the Plumecoq experimental station of the Comité Interprofessionel du Vin de Champagne (CIVC) in Epernay (France). Samples were collected at three different time points corresponding to 7-days before theoretical harvest (TH-7), theoretical harvest (TH) and 10-days after theoretical harvest (TH+10) as defined by the viticulturists of CIVC. For each date, three types of samples were collected separately along a grapevine row located in the middle of the plot. Twelve whole bunches were collected and pooled along this row, except on the 3-4 first vine stocks on both sides of the row, in order to minimize differences brought about by phytosanitary treatments, sun exposure or bunch size. The most representative berry class, based on density (i.e. total soluble solids), of a given harvest date was selected for further characterization. Density was estimated by flotation of a thousand berries into a range of NaCl solutions, each having a decrease in salinity of 10g/L NaCl (from 200 to 70g/L NaCl). A representative sample of 100 sorted berries was squeezed for measurements of total soluble solids (°BRIX) and potential alcohol degree (using a hand-held refractometer). Then, about twenty-five berries belonging to the most representative berry class were pooled. They will be referred to as densimetrically sorted berries (DSB). The twelve whole bunches (WBB) and the densimetrically sorted berries (DSB) were frozen immediately in liquid nitrogen and stored at -80°C until further use. All remaining bunches of the row were then collected and used for microvinifications and biochemical analysis.

#### - Samples from controlled environment rooms

Grapevine berries samples were taken from *Vitis vinifera *L. cv Cabernet Sauvignon rooted cuttings grown in controlled environment rooms with 16 h days and temperature regimes of either 30°C days and 25°C nights or 20°C days and 15°C nights. The rooted cuttings were encouraged to set fruit by removing leaves from emerging buds as described by Mullins [93], and only one bunch was allowed to develop on each vine. The progression of berry development and ripening was followed by measuring berry volume on a random selection of 100 berries as outlined in Boss et al. [24] and by scoring the percentage of colored berries at weekly intervals. A random sample of 50 berries were collected from either temperature treatment at fortnightly intervals starting from when the berries first showed signs of color change, and continuing for a further 10 weeks for the 30°C day and 25°C night treated berries and a further eight weeks for the cooler treatment. °BRIX measurements were made on these 50 berries using a RFM710 digital refractometer (Bellingham Stanley, Tunbridge Wells, Kent, UK) before they were frozen in liquid N_2 _and stored at -80°C pending further use.

### Determination of physiological parameters

In order to assess the evolution of Chardonnay berry ripening and to correlate it with changes in transcriptomic profiles, berry weight, total soluble solids (°BRIX), potential alcohol content (% vol) were evaluated at the three stages used for sampling. Total soluble solids (°BRIX) were measured in grapevine juice, obtained by pressing fresh berries with a small hand-crank press, using a hand held refractometer. The potential alcohol content was estimated from total soluble solids (°BRIX).

### Microvinification assays and determination of wine physicochemical parameters

Microvinification assays were performed from 160 kg of Chardonnay grapes following the traditional wine-making methods of CIVC. The decanted must and base wines were analyzed for conventional parameters according to the recommendations of the International Organization of Vine and Wine (OIV) described in the Compendium of international methods of wine and must analysis [94].

### Sensory analysis

Sensory evaluations were performed to determine whether there were significant differences between base wines made with bunches harvested during the 2005 and 2006 growing seasons at the TH-7, TH and TH+10 stages respectively. All evaluations were carried out at the CIVC in Epernay (Champagne, France) using standard wine-tasting procedures. A triangular test was designed to figure out the effects of harvest date on base wine for each vintage studied. All the triangle tests performed in this study were carried out in accordance with the ISO standard ISO 4120:2004; criteria for significant detection of the effects of harvest date were based on binomial distribution tables. The results were considered significant for α ≤ 0.05. The tasting panel was composed of 13 professional tasters used to Champagne tasting panels. A minimum number of 8 correct responses was needed to conclude that a perceptible difference exists between the tested wines. The base wine used for Champagne tasting is a still wine. The sensory qualities of base wines were evaluated around 8 months after the harvest date.

### RNA extraction

Total RNA from Chardonnay berries was isolated as previously described by Reid et al. [95]. Pedicel and seeds of each berry were removed before grinding in liquid nitrogen. Total RNA was subjected to DNA digestion with 5 units of RNase-free DNase I (Promega) for 1 h at 37°C. RNA content was measured at 260 mm with a spectrophotometer (GeneQuant™ Pro, GE Healthcare, Pessac alouette, France) and visualized by electrophoresis on 1.5% agarose gels.

Total RNA extractions from Cabernet Sauvignon cultivar were conducted on the berries using the method of Boss et al. [24], and further purified to remove genomic DNA as outlined in D'Onofrio et al. [96].

### Microarray Analysis

#### - Probe synthesis, hybridization and data acquisition

The *Vitis vinifera *microarray slides used in this study contain a set of 70-mer oligonucleotides (Operon, USA; Array-Ready Oligo Set™ for the Grape Genome, Version 1.0) representing 14,562 unigenes [11]. A total of 16 two-color microarrays were used to compare TH-7 and TH+10 timepoints from all samples (WBB 2005, WBB 2006, DSB 2005, DSB 2006) to their respective TH timepoint. A dye-swap was done for each comparison.

The Amino Allyl MessageAmp™ II aRNA Amplification Kit (Ambion, Huntingdon, UK) was used according to the manufacturer's recommendations for probe labeling. Probe assembly was performed using 600 picomol of Cy3- and Cy5-labeled aRNA. The pooled Cy3- and Cy5-labeled aRNAs were then concentrated on Microcon YM-30 columns (Amicon Bioseparations, Millipore, Molsheim, France) and mixed with 90 μL of hybridization solution containing 1:1 (v:v) formamide (5X SSC, 0.25% SDS, 5X Denhardt's solution, and 1 mg/mL denatured salmon sperm DNA. Prior to hybridization, the DNA was UV-crosslinked on the microarray chips in a Stratalinker by exposure to 100 MJ of UV light. Following crosslinking, the slides were chemically blocked by soaking them gently twice with up- and down- movement for 1 min in 0.2% SDS. Air dried slides were hybridized in an automatic hybridization station HS 4800 (Tecan, Trappes, France) with a washing prerun in 1X SSC, 0.1% SDS, for 1 min. The probe solution was boiled for 1 min at 100°C, cooled on ice for 2 min, stabilized at 37°C for 5 min and then injected into the hybridization chamber. Slides were incubated at 37°C for 16 h, with medium agitation, and then washed sequentially at 30°C in 1X SSC, 0.1% SDS for 1 min, this step was repeated three times, in 0.1X SSC, 0.1% SDS for 1 min, three times, and finally in 0.1X SSC for 30 s. Slides were dried in the hybridization station for 3 min, with 2.7 bars of nitrogen gas. Microarray slides were scanned with a Genepix 4000 B fluorescence scanner (Axon Instruments, Foster City, CA, USA) using Genepix 4.0 image acquisition software with photomultiplier tube voltage adjusted to 400 V for Cy3 (532 nm) and 460 V for Cy5 (635 nm).

#### - Microarray data processing and bioinformatic analysis

Spot flagging was done first by Genepix 4.0 (missing spots) and then by visual inspection of the images to exclude the abnormal spots (saturation and heterogeneity). Integrated pixel intensity values for each spot were calculated by using Genepix 4.0 software and saved in tab-delimited format. Median intensity values were normalized with background subtraction by a global lowess method followed by a print-tip median method. Differentially expressed genes were identified with the R/Bioconductor package Limma [97] using linear models and by taking into account technical (dye-swaps) and biological (years) replicates to assess the following contrasts: WBB TH vs TH-7, WBB TH+10 vs TH, DSB TH vs TH-7 and DSB TH+10 vs TH. For each hybridization, selection of differentially expressed clones was performed by filtering in order to include genes that were up-or down-regulated 1.75-fold at least (p-value < 0.05).

The 70-mer microarray oligonucleotides were based on the transcripts of the VvGI (*Vitis vinifera *Gene Index). The oligonucleotides were linked to the latest version of the VvGI, version 7.0, April 17, 2010 (http://compbio.dfci.harvard.edu/tgi/cgi-bin/tgi/gimain.pl?gudb=grape) and to the predicted genes of the 12X grapevine genomic sequence (http://www.genoscope.cns.fr/externe/GenomeBrowser/Vitis/) using a BLAST (blastn) program: all full length hits were selected. For each differentially expressed gene, the mRNA number was searched using a BLAST (blastn) program against all available mRNA sequences in NCBI database. The data are available in ArrayExpress (http://www.ebi.ac.uk/arrayExpress) under the accession E-MTAB-481.

### Real-Time PCR analysis

Expression analysis from Chardonnay cultivar was performed by qPCR according to Terrier et al. [98]. A triplicate reverse transcription was performed on 500 ng of total RNA from each development stage (RNA samples obtained in 2005 and 2006) using the Superscript II RT kit (Invitrogen, Fischerbioblock, Illkirch, France) according to the manufacturer's instructions in a final volume of 20 μL. Specific annealing of the oligonucleotides was controlled on dissociation kinetics performed at the end of each PCR run. The PCR was performed in triplicate on 1 μL cDNA from each sample, using a model 7300 Sequence Detection System (Applied Biosystems, Warrington, UK) and the Power SYBR-Green PCR Master kit (Applied Biosystems Applera France, Courtaboeuf, France). PCR conditions used consited of an initial denaturation step at 50°C for 2 min, 95°C for 10 min followed by 40 cycles of 95°C for 15 sec, 60°C for 1 min. All qPCR experiments were conducted in triplicate using primers designed to each gene of interest. The PCR primer combinations for each gene were as follows: *VvCCD4a *(XM_002268368): forward 5'-CCACACAGCCTTCACTCTCA-3', reverse 5'-AGGGCCTTTTTGAGAAGCAT-3'. *VvValCS *(FJ696653/AY561843): forward 5'-CGTGTATTGCCTTGTGGAAG-3', reverse 5'-TATGTGTCCCCTTGCCGTAT-3'. Relative fold differences were calculated based on the comparative Ct method using the *EF1-α *as an internal standard. To demonstrate that the efficiencies of the different gene primers were approximately equal, the absolute value of the slope of log input amount versus ΔCt was calculated as previously described [98]. To determine relative fold differences for each sample in each experiment, the Ct value for all the genes was normalized to the Ct value for *EF1-α *(control gene) and was calculated relative to a calibrator using the formula 2^-ΔΔCt ^[99].

Gene expression analysis during Cabernet Sauvignon berry development was performed by qPCR using 1 μg of total RNA. cDNA was synthesised using 1 μg of RNA using the SuperScript III first-strand cDNA synthesis kit (Invitrogen, San Diego, CA, USA) in a final volume of 20 μL, and this was diluted to 200 μL before use in the qPCR reaction mixes. qPCR was conducted using a Rotor-Gene 2000 (version 4.2) real-time cycler (Corbett Life Science, Sydney, Australia) and FastStart SYBR Green Master (Roche Diagnostics GmbH, Mannheim, Germany). The reaction mixes contained 70 nM of each primer, 2 μL of cDNA (1:10 dilution of the synthesis reaction), and 1 × FastStart SYBR Green Master mix in a final volume of 15 μL. PCR conditions used consisted of an initial denaturation step at 95°C for 15 min followed by 45 cycles of 95°C for 20 sec, 58°C for 20 sec and 72°C for 20 sec. All qPCR experiments were conducted in three technical replicates using primers designed to each gene of interest (Additional file 3, Table S3). Primer products from the qPCR reactions were analyzed by electrophoresis and melt curves to ensure that they amplified a product of one size and were sequenced to ensure that they matched the gene target. Quantification was obtained by plotting the Ct values from the berry cDNA samples against a linear calibration curve obtained from the Ct values of serially diluted cDNA of the target gene. The expression values were calculated using the standard curves for each gene. These were normalized to the mean relative expression values obtained using three reference genes (*Ubiquitin*, *Actin *and *EF1-α*) in the respective cDNA samples again using a calibration curve calculated from Ct values. Expression levels are presented relative to the lowest level of expression detected in any sample for each gene.

## List of abbreviations

bZIP: basic leucine zipper; BW: base wine; CCD: carotenoid cleavage dioxygenase; CIVC: Comité Interprofessionnel du Vin de Champagne; DIR-like: dirigent-like; DMU: decanted must; DSB: densimetrically sorted berries; EXP: expansin; FT/TFL: FLOWERING LOCUS T/TERMINAL FLOWER; GA: gibberellic acid; GolS: galactinol synthase; GRIP: grape ripening-induced protein; GT: glycosyl transferase; HK: histidine kinase; HKR: histidine kinase receptor; HR: hypersensitive response; IAA: indole-3-acetic acid; LEA: late embryogenesis abundant protein; LRR: leucine-rich repeat; OIV: International Organization of Vine and Wine; PAL: phenylalanine ammonia-lyase; PG: polygalacturonase; PL: pectate lyase; PR: pathogenesis-related protein; RALF: rapid alkalinisation factor; RFOs: raffinose family oligosaccharides; SAMT: S-adenosyl-L-methionine:salicylic acid carboxyl methyltransferase; SAR: systemic acquired resistance; SE: standard error; smHSP: small molecular weight heat shock protein; TCP: Teosinte Branched1; Cycloidea and PCF; TH: theoretical harvest; TH-7: 7-days before theoretical harvest; TH+10: 10-days after theoretical harvest; TIP: tonoplast intrinsic protein; V-ATPase: Vacuolar H+-transporting adenosine triphosphatase; V-PPase: Vacuolar H+-translocating inorganic pyrophosphatase; ValCS: (+)-valencene synthase; Vv: *Vitis vinifera*; VvGI: *Vitis vinifera *Gene index; WBB: whole bunch berries; XTH: xyloglucan endotransglycosylase/hydrolase.

## Authors' contributions

SG, RF and CC conducted the research and designed the experiments on vineyard samples. SG analyzed the data and drafted the manuscript together with SD. CK performed the microarray statistical analyses. NT and JDD contributed to acquisition of gene expression data. CD conducted the growth room experiments, berry measurements and sampling. PKB assisted in the growth room experiments and gene expression analyses, substantially edited the manuscript and participated in data interpretation and organization. SD designed and supervised the project. SD and DM coordinated the ADAR project in which this work was managed. All authors read, edited and approved the final manuscript.

## Supplementary Material

Additional file 1 **Supplementary Table S1**. Table S1. Differentially expressed genes (P < 0.05, ≥1.75-fold) of unknown function in Chardonnay grapevine berries between theoretical harvest date (TH) and one week before (TH-7) of the 2005 and 2006 growing seasons. Ratio values are presented as log2. DSB, densimetrically sorted berries; TH-7, 7-days before theoretical harvest; TH, theoretical harvest; WBB, whole bunch berries. Click here for file

Additional file 2 **Supplementary Table S2**. Table S2. Differentially expressed genes (P < 0.05, ≥1.75-fold) of unknown function in Chardonnay grapevine berries between 10-days after theoretical harvest (TH+10) and theoretical harvest date (TH) of the 2005 and 2006 growing seasons. Ratio values are presented as log2. DSB, densimetrically sorted berries; TH, theoretical harvest; TH+10, 10-days after harvest; WBB, whole bunch berries. Click here for file

Additional file 3 **Supplementary Table S3**. Table S3. qPCR primer sequences. Primer sequences used for Cabernet Sauvignon qPCR analysis and the mRNA accession number from which the sequence data was obtained for primer design. The mRNA number was searched using a BLAST (blastn) program against available mRNA sequences in NCBI database. Click here for file

## References

[B1] Coombe BG McCarthy MG Dynamics of grape berry growth and physiology of ripening Aust J Grape Wine Research 2000 6 131 135 10.1111/j.1755-0238.2000.tb00171.x

[B2] Conde C Silva P Fontes N Dias A Tavares R Sousa M Agasse A Delrot S Geros H Biochemical changes throughout grape berry development and fruit and wine quality Food 2007 1 1 22

[B3] Jaillon O Aury JM Noel B Policriti A Clepet C Casagrande A Choisne N Aubourg S Vitulo N Jubin C Vezzi A Legeai F Hugueney P Dasilva C Horner D Mica E Jublot D Poulain J Bruyère C Billault A Segurens B Gouyvenoux M Ugarte E Cattonaro F Anthouard V Vico V Del Fabbro C Alaux M di Gaspero G Dumas V The grapevine genome sequence suggests ancestral hexaploidization in major angiosperm phyla Nature 2007 449 463 467 10.1038/nature06148 17721507

[B4] Velasco R Zharkikh A Troggio M Cartwright DA Cestaro A Pruss D Pindo M Fitzgerald LM Vezzulli S Reid J Malacarne G Iliev D Coppola G Wardell B Micheletti D Macalma T Facci M Mitchell JT Perazzolli M Eldredge G Gatto P Oyzerski R Moretto M Gutin N Stefanini M Chen Y Segala C Davenport C Demattè L Mraz A A high quality draft consensus sequence of the genome of a heterozygous grapevine variety PLoS One 2007 2 e1326 10.1371/journal.pone.0001326 18094749 PMC2147077

[B5] Cramer GR Ergül A Grimplet J Tillett RL Tattersall EA Bohlman MC Vincent D Sonderegger J Evans J Osborne C Quilici D Schlauch KA Schooley DA Cushman JC Water and salinity stress in grapevines: early and late changes in transcript and metabolite profiles Funct Integr Genomics 2007 7 111 134 10.1007/s10142-006-0039-y 17136344

[B6] Waters DLE Holton TA Ablett EM Lee LS Henry RJ The ripening wine grape berry skin transcriptome Plant Science 2006 171 132 138 10.1016/j.plantsci.2006.03.002

[B7] Deluc LG Grimplet J Wheatley MD Tillett RL Quilici DR Osborne C Schooley DA Schlauch KA Cushman JC Cramer GR Transcriptomic and metabolite analyses of Cabernet Sauvignon grape berry development BMC Genomics 2007 22 429 10.1186/1471-2164-8-429 PMC2220006 18034876

[B8] Zenoni S Ferrarini A Giacomelli E Xumerle L Fasoli M Malerba G Bellin D Pezzotti M Delledonne M Characterization of transcriptional complexity during berry development in *Vitis vinifera *using RNA-Seq Plant Physiol 2010 152 1787 1795 10.1104/pp.109.149716 20118272 PMC2850006

[B9] Figueiredo A Fortes AM Ferreira S Sebastiana M Choi YH Sousa L Acioli-Santos B Pessoa F Verpoorte R Pais MS Transcriptional and metabolic profiling of g*rape (Vitis vinif*era L.) leaves unravel possible innate resistance against pathogenic fungi J Exp Bot 2008 59 3371 3381 10.1093/jxb/ern187 18648103

[B10] Rotter A Camps C Lohse M Kappel C Pilati S Hren M Stitt M Coutos-Thévenot P Moser C Usadel B Delrot S Gruden K Gene expression profiling in susceptible interaction of grapevine with its fungal pathogen *Eutypa lata*: extending MapMan ontology for grapevine BMC Plant Biol 2009 5 104 10.1186/1471-2229-9-104 PMC2731041 19656401

[B11] Camps C Kappel C Lecomte P Leon C Gomes E Coutos-Thevenot P Delrot S A transcriptomic study of grapevine (*Vitis vinifera *cv. Cabernet-Sauvignon) interaction with the vascular ascomycete fungus *Eutypa lata* J Exp Bot 2010 61 1719 1737 10.1093/jxb/erq040 20190040 PMC2852663

[B12] Gatto P Vrhovsek U Muth J Segala C Romualdi C Fontana P Pruefer D Stefanini M Moser C Mattivi F Velasco R Ripening and genotype control stilbene accumulation in healthy grapes J Agric Food Chem 2008 56 11773 11785 10.1021/jf8017707 19032022

[B13] Schwartz SH Tan BC Gage DA Zeevaart JA McCarty DR Specific oxidative cleavage of carotenoids by VP14 of maizes Science 1997 20 1872 1874 10.1126/science.276.5320.1872 9188535

[B14] Schwartz SH Qin X Zeevaart JA Characterization of a novel carotenoid cleavage dioxygenase from plants J Biol Chem 2001 6 25208 25211 10.1074/jbc.M102146200 11316814

[B15] Mendes-Pinto MM Carotenoid breakdown products the -norisoprenoids- in wine aroma Arch Biochem Biophys 2009 483 236 245 10.1016/j.abb.2009.01.008 19320050

[B16] Razungles A Günata Z Pinatel S Baumes RL Bayonove CL Etude quantitative de composés terpéniques, norisoprénoïques et de leurs précurseurs dans diverses variétés de raisin Sciences des Aliments 1993 13 59 72

[B17] Baumes RL Wirth J Bureau S Gunata Z Razungles A Biogeneration of C13-norisoprenoid compounds: experiments supportive for an apo-carotenoid pathway in grapevines Anal Chim Acta 2002 458 3 14 10.1016/S0003-2670(01)01589-6

[B18] Mathieu S Terrier N Procureur J Bigey F Günata Z A carotenoid cleavage dioxygenase from *Vitis vinifera *L.: functional characterization and expression during grape berry development in relation to C13-norisoprenoid accumulation J Exp Bot 2005 56 2721 2731 10.1093/jxb/eri265 16131507

[B19] Ahrazem O Trapero A Gómez MD Rubio-Moraga A Gómez-Gómez L Genomic analysis and gene structure of the plant carotenoid dioxygenase 4 family: a deeper study in *Crocus sativus *and its allies Genomics 2010 96 239 250 10.1016/j.ygeno.2010.07.003 20633636

[B20] Huang FC Molnár P Schwab W Cloning and functional characterization of carotenoid cleavage dioxygenase 4 genes J Exp Bot 2009 60 3011 3022 10.1093/jxb/erp137 19436048 PMC2718213

[B21] Ribéreau-Gayon P Dubourdieu D Donèche B Lonvaud A Handbook of Enology. Volume 1. The Microbiology of Wine and Vinifications 2006 2 Chichester: John Wiley & Sons, Ltd

[B22] Hrazdina G Parsons GF Mattick LR Physiological and biochemical events during development and maturation of grape berries Am J Enol Vitic 1984 35 220 227

[B23] Chen JY Wen PF Kong WF Pan QH Wan SB Huang WD Changes and subcellular localizations of the enzymes involved in phenylpropanoid metabolism during grape berry development J Plant Physiol 2006 163 115 127 10.1016/j.jplph.2005.07.006 16399002

[B24] Boss PK Davies C Robinson SP Analysis of the expression of anthocyanin pathway genes in developing *Vitis vinifera *L. cv Shiraz grape berries and the implications for pathway regulation Plant Physiol 1996 111 1059 1066 12226348 10.1104/pp.111.4.1059 PMC160981

[B25] Oh S Park S Han K-H Transcriptional regulation of secondary growth in *Arabidopsis thaliana* J Exp Bot 2003 54 2709 2722 10.1093/jxb/erg304 14585825

[B26] Scheible W-R Morcuende R Czechowski T Fritz C Osuna D Palacios-Rojas N Schindelasch D Thimm O Udvardi MK Stitt M Genome-wide reprogramming of primary and secondary metabolism, protein synthesis, cellular growth processes, and the regulatory infrastructure of Arabidopsis in response to nitrogen Plant Physiol 2004 136 2483 2499 10.1104/pp.104.047019 15375205 PMC523316

[B27] Sado PE Tessier D Vasseur M Elmorjani K Guillon F Saulnier L Integrating genes and phenotype: a wheat-Arabidopsis-rice glycosyltransferase database for candidate gene analyses Funct Integr Genomics 2009 9 43 58 10.1007/s10142-008-0100-0 19005709

[B28] Downie B Gurusinghe S Dahal P Thacker RR Snyder JC Nonogaki H Yim K Fukanaga K Alvarado V Bradford KJ Expression of a *GALACTINOL SYNTHASE *gene in tomato seeds is up-regulated before maturation desiccation and again after imbibition whenever radicle protrusion is prevented Plant Physiol 2003 131 1347 1359 10.1104/pp.016386 12644684 PMC166894

[B29] Taji T Ohsumi C Iuchi S Seki M Kasuga M Kobayashi M Yamaguchi-Shinozaki K Shinozaki K Important roles of drought- and cold-inducible genes for galactinol synthase in stress tolerance in *Arabidopsis thaliana* Plant J 2002 29 417 426 10.1046/j.0960-7412.2001.01227.x 11846875

[B30] Haritatos E Ayre BG Turgeon R Identification of phloem involved in assimilate loading in leaves by the activity of the galactinol synthase promoter Plant Physiol 2000 123 929 937 10.1104/pp.123.3.929 10889241 PMC59055

[B31] Wise MJ Tunnacliffe A POPP the question: what do LEA proteins do? Trends Plant Sci 2004 9 13 17 10.1016/j.tplants.2003.10.012 14729214

[B32] van Loon LC Rep M Pieterse CM Significance of inducible defense-related proteins in infected plants Annu Rev Phytopathol 2006 44 135 162 10.1146/annurev.phyto.44.070505.143425 16602946

[B33] Sels J Mathys J De Coninck BM Cammue BP De Bolle MF Plant pathogenesis-related (PR) proteins: a focus on PR peptides Plant Physiol Biochem 2008 46 941 950 10.1016/j.plaphy.2008.06.011 18674922

[B34] Ferreira RB Monteiro SS Piçarra-Pereira MA Teixeira AR Engineering grapevine for increased resistance to fungal pathogens without compromising wine stability Trends Biotechnol 2004 22 168 173 10.1016/j.tibtech.2004.02.001 15038921

[B35] Robert N Ferran J Breda C Coutos-Thévenot P Boulay M Buffard D Esnault R Molecular characterization of the incompatible interaction of *Vitis vinifera *leaves with *Pseudomonas syringae *pv. *pisi *expression of genes coding for stilbene synthase and class 10 PR protein Eur J Plant Pathol 2001 107 249 261 10.1023/A:1011241001383

[B36] Ralph SG Jancsik S Bohlmann J Dirigent proteins in conifer defense II: Extended gene discovery, phylogeny, and constitutive and stress-induced gene expression in spruce (*Picea *spp.) Phytochemistry 2007 68 1975 1991 10.1016/j.phytochem.2007.04.042 17590394

[B37] Davin LB Wang HB Crowell AL Bedgar DL Martin DM Sarkanen S Lewis NG Stereoselective bimolecular phenoxy radical coupling by an auxiliary (dirigent) protein without an active center Science 1997 275 362 366 10.1126/science.275.5298.362 8994027

[B38] Hejátko J Ryu H Kim GT Dobesová R Choi S Choi SM Soucek P Horák J Pekárová B Palme K Brzobohaty B Hwang I The histidine kinases CYTOKININ-INDEPENDENT1 and ARABIDOPSIS HISTIDINE KINASE2 and 3 regulate vascular tissue development in Arabidopsis shoots Plant Cell 2009 21 2008 2021 10.1105/tpc.109.066696 19622803 PMC2729606

[B39] Wakabayashi K Changes in cell wall polysaccharides during fruit ripening J Plant Res 2000 113 231 237 10.1007/PL00013932

[B40] Ishimaru M Smith DL Gross KC Kobayashi S Expression of three expansin genes during development and maturation of Kyoho grape berries J Plant Physiol 2007 164 1675 1682 10.1016/j.jplph.2006.07.017 17175064

[B41] Dotto MC Martínez GA Civello PM Expression of expansin genes in strawberry varieties with contrasting fruit firmness Plant Physiol Biochem 2006 44 301 307 10.1016/j.plaphy.2006.06.008 16889972

[B42] Deytieux-Belleau C Vallet A Donèche B Geny L Pectin methylesterase and polygalacturonase in the developing grape skin Plant Physiol Biochem 2008 46 638 646 10.1016/j.plaphy.2008.04.008 18513987

[B43] Sterling JD Atmodjo MA Inwood SE Kumar Kolli VS Quigley HF Hahn MG Mohnen D Functional identification of an Arabidopsis pectin biosynthetic homogalacturonan galacturonosyltransferase Proc Natl Acad Sci USA 2006 103 5236 5241 10.1073/pnas.0600120103 16540543 PMC1458824

[B44] Nunan KJ Sims IM Bacic A Robinson SP Fincher GB Changes in cell wall composition during ripening of grape berries Plant Physiol 1998 118 783 792 10.1104/pp.118.3.783 9808722 PMC34788

[B45] Rose JK Bennett AB Cooperative disassembly of the cellulose-xyloglucan network of plant cell walls: parallels between cell expansion and fruit ripening Trends Plant Sci 1999 4 176 183 10.1016/S1360-1385(99)01405-3 10322557

[B46] Yokoyama R Nishitani K A comprehensive expression analysis of all members of a gene family encoding cell-wall enzymes allowed us to predict cis-regulatory regions involved in cell-wall construction in specific organs of Arabidopsis Plant Cell Physiol 2001 42 1025 1033 10.1093/pcp/pce154 11673616

[B47] Nunan KJ Davies C Robinson SP Fincher GB Expression patterns of cell wall-modifying enzymes during grape berry development Planta 2001 214 257 264 10.1007/s004250100609 11800390

[B48] Glissant D Dédaldéchamp F Delrot S Transcriptomic analysis of grape berry softening during ripening J Int Sci Vigne Vin 2008 42 1 13

[B49] Saladié M Rose JK Cosgrove DJ Catalá C Characterization of a new xyloglucan endotransglucosylase/hydrolase (XTH) from ripening tomato fruit and implications for the diverse modes of enzymic action Plant J 2006 47 282 295 10.1111/j.1365-313X.2006.02784.x 16774648

[B50] Lu W Wang Y Jiang Y Li J Liu H Duan X Song L Differential expression of litchi XET genes in relation to fruit growth Plant Physiol Biochem 2006 44 707 713 10.1016/j.plaphy.2006.09.020 17079153

[B51] Nishiyama K Guis M Rose JK Kubo Y Bennett KA Wangjin L Kato K Ushijima K Nakano R Inaba A Bouzayen M Latche A Pech JC Bennett AB Ethylene regulation of fruit softening and cell wall disassembly in Charentais melon J Exp Bot 2007 58 1281 1290 10.1093/jxb/erl283 17308329

[B52] Hirai T Sato M Toyooka K Sun HJ Yano M Ezura H Miraculin, a taste-modifying protein is secreted into intercellular spaces in plant cells J Plant Physiol 2010 167 209 215 10.1016/j.jplph.2009.08.001 19712996

[B53] Zhang CK Lang P Dane F Ebel RC Singh NK Locy RD Dozier WA Cold acclimation induced genes of trifoliate orange (*Poncirus trifoliata*) Plant Cell Rep 2005 23 764 769 10.1007/s00299-004-0883-y 15449021

[B54] Mondego JM Duarte MP Kiyota E Martínez L de Camargo SR De Caroli FP Alves BS Guerreiro SM Oliva ML Guerreiro-Filho O Menossi M Molecular characterization of a miraculin-like gene differentially expressed during coffee development and coffee leaf miner infestation Planta 2011 233 123 137 10.1007/s00425-010-1284-9 20931223

[B55] Grimplet J Deluc LG Tillett RL Wheatley MD Schlauch KA Cramer GR Cushman JC Tissue-specific mRNA expression profiling in grape berry tissues BMC Genomics 2007 21 187 10.1186/1471-2164-8-187 PMC1925093 17584945

[B56] Fouquet R Léon C Ollat N Barrieu F Identification of grapevine aquaporins and expression analysis in developing berries Plant Cell Rep 2008 27 1541 1550 10.1007/s00299-008-0566-1 18560835

[B57] Bourgis F Roje S Nuccio ML Fisher DB Tarczynski MC Li C Herschbach C Rennenberg H Pimenta MJ Shen TL Gage DA Hanson AD S-methylmethionine plays a major role in phloem sulfur transport and is synthesized by a novel type of methyltransferase Plant Cell 1999 11 1485 1498 10449582 10.1105/tpc.11.8.1485 PMC144290

[B58] Buchner P Stuiver CE Westerman S Wirtz M Hell R Hawkesford MJ De Kok LJ Regulation of sulfate uptake and expression of sulfate transporter genes in *Brassica oleracea *as affected by atmospheric H(2)S and pedospheric sulfate nutrition Plant Physiol 2004 136 3396 3408 10.1104/pp.104.046441 15377780 PMC523398

[B59] Tatematsu K Nakabayashi K Kamiya Y Nambara E Transcription factor AtTCP14 regulates embryonic growth potential during seed germination in *Arabidopsis thaliana* Plant J 2008 53 42 52 10.1111/j.1365-313X.2007.03308.x 17953649

[B60] Lund ST Bohlmann J The molecular basis for wine grape quality--a volatile subject Science 2006 311 804 805 10.1126/science.1118962 16469915

[B61] Boido E Lloret A Medina K Fariña L Carrau F Versini G Dellacassa E Aroma composition of *Vitis vinifera *Cv. tannat: the typical red wine from Uruguay J Agric Food Chem 2003 51 5408 5413 10.1021/jf030087i 12926890

[B62] Deluc LG Quilici DR Decendit A Grimplet J Wheatley MD Schlauch KA Mérillon JM Cushman JC Cramer GR Water deficit alters differentially metabolic pathways affecting important flavor and quality traits in grape berries of Cabernet Sauvignon and Chardonnay BMC Genomics 2009 10 212 10.1186/1471-2164-10-212 19426499 PMC2701440

[B63] Lücker J Bowen P Bohlmann J *Vitis vinifera *terpenoid cyclases: functional identification of two sesquiterpene synthase cDNAs encoding (+)-valencene synthase and (-)-germacrene D synthase and expression of mono- and sesquiterpene synthases in grapevine flowers and berries Phytochemistry 2004 65 2649 2659 10.1016/j.phytochem.2004.08.017 15464152

[B64] Robinson SP Davies C Molecular biology of grape berry ripening Aust J grape Wine Research 2000 6 175 188 10.1111/j.1755-0238.2000.tb00177.x

[B65] Aziz A Gauthier A Bézier A Poinssot B Joubert JM Pugin A Heyraud A Baillieul F Elicitor and resistance-inducing activities of beta-1,4 cellodextrins in grapevine, comparison with beta-1,3 glucans and alpha-1,4 oligogalacturonides J Exp Bot 2007 58 1463 1472 10.1093/jxb/erm008 17322548

[B66] Deytieux C Geny L Lapaillerie D Claverol S Bonneu M Donèche B Proteome analysis of grape skins during ripening J Exp Bot 2007 58 1851 1862 10.1093/jxb/erm049 17426054

[B67] Roy Choudhury S Roy S Sengupta DN Characterization of cultivar differences in beta-1,3 glucanase gene expression, glucanase activity and fruit pulp softening rates during fruit ripening in three naturally occurring banana cultivars Plant Cell Rep 2009 28 1641 1653 10.1007/s00299-009-0764-5 19697038

[B68] Tattersall DB van Heeswijck R Høj PB Identification and characterization of a fruit-specific, thaumatin-like protein that accumulates at very high levels in conjunction with the onset of sugar accumulation and berry softening in grapes Plant Physiol 1997 114 759 769 10.1104/pp.114.3.759 9232867 PMC158362

[B69] Davies C Robinson SP Differential screening indicates a dramatic change in mRNA profiles during grape berry ripening. Cloning and characterization of cDNAs encoding putative cell wall and stress response proteins Plant Physiol 2000 122 803 812 10.1104/pp.122.3.803 10712544 PMC58916

[B70] Diévart A Clark SE LRR-containing receptors regulating plant development and defense Development 2004 131 251 261 14701679 10.1242/dev.00998

[B71] di Gaspero G Cipriani G Nucleotide binding site/leucine-rich repeats, Pto-like and receptor-like kinases related to disease resistance in grapevine Mol Genet Genomics 2003 269 612 623 10.1007/s00438-003-0884-5 12884009

[B72] Timperio AM Egidi MG Zolla L Proteomics applied on plant abiotic stresses: role of heat shock proteins (HSP) J Proteomics 2008 71 391 411 10.1016/j.jprot.2008.07.005 18718564

[B73] Neta-Sharir I Isaacson T Lurie S Weiss D Dual role for tomato heat shock protein 21: protecting photosystem II from oxidative stress and promoting color changes during fruit maturation Plant Cell 2005 17 1829 1838 10.1105/tpc.105.031914 15879560 PMC1143080

[B74] Medina-Escobar N Cárdenas J Muñoz-Blanco J Caballero JL Cloning and molecular characterization of a strawberry fruit ripening-related cDNA corresponding a mRNA for a low-molecular-weight heat-shock protein Plant Mol Biol 1998 36 33 42 10.1023/A:1005994800671 9484460

[B75] Carmona MJ Calonje M Martínez-Zapater JM The *FT/TFL1 *gene family in grapevine Plant Mol Biol 2007 63 637 650 10.1007/s11103-006-9113-z 17160562

[B76] Sreekantan L Thomas MR *VvFT *and *VvMADS8*, the grapevine homologues of the floral integrators *FT *and *SOC1*, have unique expression patterns in grapevine and hasten flowering in *Arabidopsis* Funct Plant Biol 2006 33 1129 1139 10.1071/FP06144 32689323

[B77] Germain H Chevalier E Caron S Matton DP Characterization of five RALF-like genes from *Solanum chacoense *provides support for a developmental role in plants Planta 2005 220 447 454 10.1007/s00425-004-1352-0 15293049

[B78] Marín-Rodríguez MC Orchard J Seymour GB Pectate lyases, cell wall degradation and fruit softening J Exp Bot 2002 53 2115 2119 10.1093/jxb/erf089 12324535

[B79] Chourasia A Sane V Nath P Differential expression of pectate lyase during ethylene-induced postharvest softening of mango (*Mangifera indica *var. Dashehari) Physiol Plant 2006 128 546 555 10.1111/j.1399-3054.2006.00752.x

[B80] Benítez-Burraco A Blanco-Portales R Redondo-Nevado J Bellido ML Moyano E Caballero JL Muñoz-Blanco J Cloning and characterization of two ripening-related strawberry (*Fragaria × ananassa *cv. Chandler) pectate lyase genes J Exp Bot 2003 54 633 645 10.1093/jxb/erg065 12554706

[B81] Davies C Boss PK Robinson SP Treatment of Grape Berries, a Nonclimacteric Fruit with a Synthetic Auxin, Retards Ripening and Alters the Expression of Developmentally Regulated Genes Plant Physiol 1997 115 1155 1161 12223864 10.1104/pp.115.3.1155 PMC158580

[B82] Böttcher C Keyzers RA Boss PK Davies C Sequestration of auxin by the indole-3-acetic acid-amido synthetase GH3-1 in grape berry (*Vitis vinifera *L.) and the proposed role of auxin conjugation during ripening J Exp Bot 2010 61 3615 3625 10.1093/jxb/erq174 20581124

[B83] Song J Chai Y Pang Y Zuo K Fei J Liu X Sun X Tang K Isolation and characterization of an IAA-responsive gene from *Gossypium barbadense *L DNA Seq 2004 15 71 76 10.1080/10425170310001652183 15354358

[B84] Yang X Tu L Zhu L Fu L Min L Zhang X Expression profile analysis of genes involved in cell wall regeneration during protoplast culture in cotton by suppression subtractive hybridization and macroarray J Exp Bot 2008 59 3661 3674 10.1093/jxb/ern214 18775953 PMC2561149

[B85] Hedden P Phillips AL Gibberellin metabolism: new insights revealed by the genes Trends Plant Sci 2000 5 523 530 10.1016/S1360-1385(00)01790-8 11120474

[B86] Boss PK Thomas MR Association of dwarfism and floral induction with a grape 'green revolution' mutation Nature 2002 416 847 850 10.1038/416847a 11976683

[B87] Terrier N Sauvage FX Ageorges A Romieu C Changes in acidity and in proton transport at the tonoplast of grape berries during development Planta 2001 213 20 28 10.1007/s004250000472 11523652

[B88] Venter M Groenewald JH Botha FC Sequence analysis and transcriptional profiling of two vacuolar H+ -pyrophosphatase isoforms in *Vitis vinifera* J Plant Res 2006 119 469 478 10.1007/s10265-006-0009-4 16924561

[B89] Tyerman SD Tilbrook J Pardo C Kotula L Sullivan W Steudle E Direct measurements of hydraulic properties in developing berries of *Vitis vinifera *L. cv. Shiraz and Chardonnay Aust J Grape Wine Res 2004 10 170 181

[B90] Ross JR Nam KH D'Auria JC Pichersky E S-Adenosyl-L-methionine:salicylic acid carboxyl methyltransferase, an enzyme involved in floral scent production and plant defense, represents a new class of plant methyltransferases Arch Biochem Biophys 1999 367 9 16 10.1006/abbi.1999.1255 10375393

[B91] Negre F Kolosova N Knoll J Kish CM Dudareva N Novel S-adenosyl-L-methionine:salicylic acid carboxyl methyltransferase, an enzyme responsible for biosynthesis of methyl salicylate and methyl benzoate, is not involved in floral scent production in snapdragon flowers Arch Biochem Biophys 2002 406 261 270 10.1016/S0003-9861(02)00458-7 12361714

[B92] Chuine I Yiou P Viovy N Seguin B Daux V Le Roy Ladurie E Historical phenology: grape ripening as a past climate indicator Nature 2004 432 289 290 10.1038/432289a 15549085

[B93] Mullins MG Test plants for investigations of the physiology of fruiting in *Vitis vinifera *L Nature 1966 209 419 420 10.1038/209419a0

[B94] Organization of Vine and Wine Compendium of international methods of wine and must analysis. Paris 2006

[B95] Reid KE Olsson N Schlosser J Peng F Lund ST An optimized grapevine RNA isolation procedure and statistical determination of reference genes for real-time RT-PCR during berry development BMC Plant Biol 2006 6 27 10.1186/1471-2229-6-27 17105665 PMC1654153

[B96] D'Onofrio C Cox A Davies C Boss PK Induction of secondary metabolism in grape cell cultures by jasmonates Funct Plant Biol 2009 36 323 338 10.1071/FP08280 32688650

[B97] Smyth GK Linear models and empirical Bayes methods for assessing differential expression in microarray experiments Statistical Applications in Genetics and Molecular Biology 2004 3 Article 3 10.2202/1544-6115.1027 16646809

[B98] Terrier N Glissant D Grimplet J Barrieu F Abbal P Couture C Ageorges A Atanassova R Léon C Renaudin JP Dédaldéchamp F Romieu C Delrot S Hamdi S Isogene specific oligo arrays reveal multifaceted changes in gene expression during grape berry (*Vitis vinifera *L.) development Planta 2005 222 832 847 10.1007/s00425-005-0017-y 16151847

[B99] Livak KJ Schmittgen TD Analysis of relative gene expression data using real-time quantitative PCR and the 2(-Delta Delta C(T)) Method Methods 2001 25 402 408 10.1006/meth.2001.1262 11846609

