# Supplementary material for: Transcriptional analysis of late ripening stages of grapevine berry
Source: BMC Plant Biol. 2011 Nov 18;11:165. doi: 10.1186/1471-2229-11-165 (PMC3233516; doi:10.1186/1471-2229-11-165)
Supplement: Additional file 1 — Supplementary Table S1. Table S1. Differentially expressed genes (P < 0.05, ≥1.75-fold) of unknown function in Chardonnay grapevine berries between theoretical harvest date (TH) and one week before (TH-7) of the 2005 and 2006 growing seasons. Ratio values are presented as log2. DSB, densimetrically sorted berries; TH-7, 7-days before theoretical harvest; TH, theoretical harvest; WBB, whole bunch berries. [file 1471-2229-11-165-S1.DOC]

| Table S1. Differentially expressed genes (P < 0.05, ≥1.75-fold) of unknown function in Chardonnay grapevine berries between theoretical harvest date (TH) and one week before (TH-7) of the 2005 and 2006 growing seasons. | | | | | | | |
| --- | --- | --- | --- | --- | --- | --- | --- |
| Grape Microarray Accession Number (Vv_#) | Grape Nucleotide Accession Number (mRNA) | Grape Gene Accession Number (GSVIVT#) | Most Homologous *Arabidopsis* Sequence | WBB average ratio | p-value | DSB average ratio | p-value |
| Vv_10003148 | XM_002280849 | GSVIVT01019636001 | At1g64640 | -1.087 | 0.00017 | -1.174 | 0.00017 |
| Vv_10004638 | EE090683 | GSVIVT01033335001 | - | -1.015 | 0.00063 | -0.998 | 0.00033 |
| Vv_10005034 | BQ794653 | GSVIVT01032930001 | - | -1.09 | 0.00073 | -1.147 | 0.00031 |
| Vv_10006335 | XM_002281318 | GSVIVT01016428001 | At3g46230 | -0.906 | 0.00042 | -0.852 | 0.0009 |
| Vv_10006849 | XM_002274475 | GSVIVT01034396001 | At5g23350 | -0.847 | 0.00054 | -0.868 | 0.00074 |
| Vv_10008539 | XM_002285741 | GSVIVT01009066001 | At4g08950 | -1.133 | 0.00037 | -0.925 | 0.00092 |
| Vv_10009130 | XM_002283124 | GSVIVT01033335001 | - | -2.311 | 2.00E-05 | -2.07 | 4.00E-05 |
| Vv_10009159 | XM_002283088 | - | - | -1.128 | 0.00082 | -0.852 | 0.00088 |
| Vv_10009224 | XM_002273452 | GSVIVT01022370001 | At3g05270 | -1.201 | 0.00063 | -1.183 | 0.00014 |
| Vv_10009951 | CD799845 | GSVIVT01009322001 | - | -1.206 | 0.00069 | -0.942 | 0.00273 |
| Vv_10010811 | CB340926 | - | - | 3.55 | 0.00554 | 7.965 | 0.00268 |
| Vv_10011725 | XM_002283788 | GSVIVT01009614001 | - | 1.319 | 0.00011 | 0.984 | 0.00355 |
| Vv_10011954 | XM_002283772 | GSVIVT01025834001 | At4g24380 | 1.156 | 0.00022 | 0.889 | 0.00032 |
| Vv_10012270 | BQ794851 | GSVIVT01038661001 | - | -0.801 | 0.00397 | -0.799 | 0.0023 |
| Vv_10012395 | XM_002271414 | - | - | 1.102 | 0.00015 | 1.237 | 0.00014 |
| Vv_10013006 | CA810951 | - | - | -0.845 | 0.00062 | -0.873 | 0.0006 |
| Vv_10013100 | XM_002277387 | GSVIVT01033335001 | - | -0.971 | 0.00145 | -0.983 | 0.00125 |
| Vv_10013215 | BQ794654 | GSVIVT01032930001 | - | -1.119 | 0.00153 | -1.176 | 0.00069 |
| Vv_10013488 | XM_002283088 | - | - | -1.244 | 0.00066 | -0.892 | 0.00122 |
| Vv_10013891 | XM_002279597 | GSVIVT01017099001 | At1g79910 | -0.995 | 0.00239 | -1.026 | 0.00156 |
| Ratio values are presented as log2. DSB, densimetrically sorted berries; TH-7, 7-days before theoretical harvest; TH, theoretical harvest; WBB, whole bunch berries. | | | | | | | |
