# Supplementary material for: Transcriptional analysis of late ripening stages of grapevine berry
Source: BMC Plant Biol. 2011 Nov 18;11:165. doi: 10.1186/1471-2229-11-165 (PMC3233516; doi:10.1186/1471-2229-11-165)
Supplement: Additional file 2 — Supplementary Table S2. Table S2. Differentially expressed genes (P < 0.05, ≥1.75-fold) of unknown function in Chardonnay grapevine berries between 10-days after theoretical harvest (TH+10) and theoretical harvest date (TH) of the 2005 and 2006 growing seasons. Ratio values are presented as log2. DSB, densimetrically sorted berries; TH, theoretical harvest; TH+10, 10-days after harvest; WBB, whole bunch berries. [file 1471-2229-11-165-S2.DOC]

| Table S2. Differentially expressed genes (P < 0.05, ≥1.75-fold) of unknown function in Chardonnay grapevine berries between 10-days after theoretical harvest (TH+10) and theoretical harvest date (TH) of the 2005 and 2006 growing seasons. | | | | | | | |
| --- | --- | --- | --- | --- | --- | --- | --- |
| Grape Microarray Accession Number (Vv_#) | Grape Nucleotide Accession Number (mRNA) | Grape Gene Accession Number (GSVIVT#) | Most Homologous *Arabidopsis* Sequence | WBB average ratio | p-value | DSB average ratio | p-value |
| Vv_10007815 | CD719011 | GSVIVT01021149001 | At1g58170 | -1.016 | 0.00044 | -0.91 | 0.00084 |
| Vv_10009030 | XM_002268045 | GSVIVT01021261001 | At3g14870 | -0.811 | 0.00241 | -1.102 | 0.00022 |
| Vv_10010268 | XM_002285646 | GSVIVT01025391001 | - | 1.685 | 0.00236 | 1.465 | 0.00075 |
| Vv_10010851 | CD008061 | GSVIVT01035428001 | - | -1.012 | 0.01348 | -0.965 | 0.00946 |
| Vv_10011056 | CA814196 | - | - | -1.018 | 0.00786 | -1.112 | 0.01018 |
| Vv_10011367 | CD710731 | - | - | 1.511 | 4.00E-04 | 1.152 | 0.00018 |
| Vv_10011993 | CB341842 | GSVIVT01001657001 | - | 1.69 | 0.00032 | 1.888 | 0.00339 |
| Vv_10013513 | CD009042 | GSVIVT01021388001 |  | 0.995 | 0.00169 | 1.127 | 0.00018 |
| Vv_10013592 | CF605569 | GSVIVT01037841001 | - | 0.828 | 0.00371 | 0.992 | 0.00124 |
| Vv_10000280 | XM_002264134 | - | - | -1.092 | 0.00298 | -1.319 | 0.00127 |
| Vv_10011345 | XM_002263043 | - | - | -1.227 | 1.00E-04 | -1.465 | 0.00008 |
| Vv_10000420 | CA814462 | GSVIVT01016697001 | - | -0.941 | 0.00415 | -1.086 | 0.01179 |
| Vv_10000680 | XM_002268392 | GSVIVT01005061001 | At3g50440 | 1.595 | 6.00E-05 | 1.025 | 0.00048 |
| Vv_10003589 | CB920638 | GSVIVT01031160001 | - | -0.839 | 0.00058 | -1.324 | 0.00015 |
| Vv_10001062 | XM_002265499 | - | - | 1.199 | 0.01166 | 0.856 | 0.01091 |
| Vv_10010515 | CB979613 | GSVIVT01010993001 |  | 0.946 | 0.00063 | 1.399 | 0.00011 |
| Ratio values are presented as log2. DSB, densimetrically sorted berries; TH, theoretical harvest; TH+10, 10-days after harvest; WBB, whole bunch berries. | | | | | | | |
