# Supplementary material for: Transcriptional analysis of late ripening stages of grapevine berry
Source: BMC Plant Biol. 2011 Nov 18;11:165. doi: 10.1186/1471-2229-11-165 (PMC3233516; doi:10.1186/1471-2229-11-165)
Supplement: Additional file 3 — Supplementary Table S3. Table S3. qPCR primer sequences. Primer sequences used for Cabernet Sauvignon qPCR analysis and the mRNA accession number from which the sequence data was obtained for primer design. The mRNA number was searched using a BLAST (blastn) program against available mRNA sequences in NCBI database. [file 1471-2229-11-165-S3.DOC]

Table S3. qPCR primer sequences.

| Gene | mRNA accession number | Forward Primer | Reverse Primer |
| --- | --- | --- | --- |
| *VvCCD4a* | XM_002268368 | 5´-CGCACTGGCCGTATAATTT-3´ | 5´-CACAGGGCCTTTTTGAGAA-3´ |
| *VvDIR-like* | XM_002285641 | 5´-CAATGGAGATGGCACTATTGAA-3´ | 5´-TCAAATGAAGTAAGAGCCACCTAA-3´ |
| *VvGolS* | XM_002262669 | 5´-AAGCTTGAAGGATCATGACG-3´ | 5´-TCCCAACAACAAACTGCTCA-3´ |
| *VvHKR* | FJ822975 | 5´-TTCCTTCATCGCTGAGTGGT-3´ | 5´-GCAGAATGGTCGAAAAGCTC-3´ |
| *VvLEA1* | XM_002283966 | 5´-AGCAATCTAAGGACGCTCCA-3´ | 5´-ACACGTGCAATCTCCTCCTC-3´ |
| *VvPAL2* | AB015871 | 5´-TGCTGACTGGTGAAAAGGTG-3´ | 5´-AATGGAAAATAAAGAGTGTGGAAGG-3´ |
| *VvSAMT* | XM_002262640 | 5´-CTGCCCATCAGATGCTCATA-3´ | 5´-GCTTATTTAGTAAGACGTACATACG-3´ |
| *VvValCS* | FJ696653/AY561843 | 5´-TGGGAAAGTGATGAGGGATAA-3´ | 5´-TGCCCCTTGCCGTATTACAA-3´ |
| *Actin* | XM_002282480 | 5´-GCATCCCTCAGCACCTTCCA-3´ | 5´-AACCCCACCTCAACACATCTCC-3´ |
| *EF1-α* | XM_002264364 | 5´-CGGGCAAGAGATACCTCAAT-3´ | 5´-AGAGCCTCTCCCTCAAAAGG-3´ |
| *Ubiquitin* | XM_002273532 | 5´-GAGTATCAAAACAAAAGC-3´ | 5´-AGTAGATGACTGCATTGG-3´ |
| Primer sequences used for Cabernet Sauvignon qPCR analysis and the mRNA accession number from which the sequence data was obtained for primer design. The mRNA number was searched using a BLAST (blastn) program against available mRNA sequences in NCBI database. | | | |
